# *Drosophila* Tet Is Required for Maintaining Glial Homeostasis in Developing and Adult Fly Brains

**DOI:** 10.1523/ENEURO.0418-21.2022

**Published:** 2022-04-22

**Authors:** Felice Frey, Jawdat Sandakly, Mirna Ghannam, Caren Doueiry, Fredrik Hugosson, Johannes Berlandi, Joy N. Ismail, Tenzin Gayden, Martin Hasselblatt, Nada Jabado, Margret Shirinian

**Affiliations:** 1 Department of Experimental Pathology, Immunology and Microbiology, Faculty of Medicine, American University of Beirut, Beirut 1107 2020, Lebanon; 2 Department of Human Genetics, McGill University, Montreal, Quebec H4A 3J1, Canada; 3 The Whitney Laboratory for Marine Bioscience, University of Florida, St. Augustine, Florida, 32080-8610; 4 Institute of Neuropathology, University Hospital Münster, Münster 48149, Germany

**Keywords:** 6mA DNA demethylation, brain tumor, *Drosophila* brain, *Drosophila* Ten-eleven translocation (Tet), human TET *Drosophila* model, optic chiasm glia

## Abstract

Ten-eleven translocation (TET) proteins are crucial epigenetic regulators highly conserved in multicellular organisms. TETs’ enzymatic function in demethylating 5-methyl cytosine in DNA is required for proper development and TETs are frequently mutated in cancer. Recently, *Drosophila melanogaster* Tet (dTet) was shown to be highly expressed in developing fly brains and discovered to play an important role in brain and muscle development as well as fly behavior. Furthermore, dTet was shown to have different substrate specificity compared with mammals. However, the exact role dTet plays in glial cells and how ectopic TET expression in glial cells contributes to tumorigenesis and glioma is still not clear. Here, we report a novel role for dTet specifically in glial cell organization and number. We show that loss of dTet affects the organization of a specific glia population in the optic lobe, the “optic chiasm” glia. Additionally, we find irregularities in axon patterns in the ventral nerve cord (VNC) both, in the midline and longitudinal axons. These morphologic glia and axonal defects were accompanied by locomotor defects in developing larvae escalating to immobility in adult flies. Furthermore, glia homeostasis was disturbed in dTet-deficient brains manifesting in gain of glial cell numbers and increased proliferation. Finally, we establish a *Drosophila* model to understand the impact of human TET3 in glia and find that ectopic expression of hTET3 in dTet-expressing cells causes glia expansion in larval brains and affects sleep/rest behavior and the circadian clock in adult flies.

## Significance Statement

TET proteins are important regulators of cellular identity and loss of function is associated with cancer onset. Recently, several studies embarked on understanding how this single demethylase affects brain development and regulates transcription. This study reports a yet undescribed role for dTet in normal glial homeostasis, proper arrangement in optic chiasm glia and behavior. We show that loss of dTet is accompanied by a highly significant increase in glia cell numbers in the optic lobe region of *Drosophila* brains that coincides with changes in hippo pathway activation in larval brains. Finally, we establish a human TET3 *Drosophila* model, and show that ectopic expression of TET3 results in deregulation of glia proliferation in the optic lobe and affects fly survival and circadian rhythm.

## Introduction

DNA methylation plays a central role in numerous processes including development, X-chromosome inactivation as well as transcriptional silencing and imbalance in DNA methylation patterns are a hallmark of cancer cells. The Ten-eleven translocation (TET) protein family consists of three members that play a key role in active DNA demethylation in mammals (Tahiliani et al., 2009). TET proteins are important epigenetic regulators involved in controlling cellular identity and highly conserved in multicellular organisms. Loss of TET function is associated with cancer onset and progression (Scourzic et al., 2015; Rasmussen and Helin, 2016). TET proteins are iron (II) and α-ketoglutarate (2-OG) dependent dioxygenases that catalyze the successive oxidation of 5-methylcytosine (5mC) to 5-hydroxymethylcytosine (5hmC) and further oxidized derivatives (Tahiliani et al., 2009; Ito et al., 2011). Enzymes involved in DNA repair can then remove these oxidized methyl-cytosines by triggering base excision repair, ultimately replacing the modified cytosines with unmodified ones (He et al., 2011). TET-mediated demethylation is not only crucial for regulation of DNA methylation fidelity, but has also been suggested to play a role in ensuring timely gene expression during development and differentiation (Rudenko et al., 2013; Rawłuszko-Wieczorek et al., 2015). Importantly, 5hmC the product of 5-methylcytosine conversion by TET proteins is not only a mere intermediate of DNA demethylation, but has emerged as a novel and stable epigenetic mark capable of influencing chromatin structure and involved in gene regulation and development (Wossidlo et al., 2011; Bocker et al., 2012).

Since epigenetic modifications can be rapidly regulated to modulate gene expression and adjust expression levels in an environmental-dependent way, TET proteins have been suggested as potential mediators of dynamic processes such as neuronal plasticity and behavior. TET family dioxygenases, with their ability to demethylate 5mC to 5hmC, have been linked to neuronal plasticity and long-term behaviors, such as cognition (Antunes et al., 2019). Furthermore, 5hmC is suggested to play an important role in the epigenetic regulation of transcription in the brain, mediating brain development and maintenance of adult CNS function (Al-Mahdawi et al., 2014).

Similar to mammalian TETs, *Drosophila* Tet (dTet) has been shown to regulate expression of genes involved in fly neurodevelopment and neuronal function, specifically important for behavior as well as learning and memory (Yao et al., 2018). While dTet expression in fly brains has been mainly linked to neuronal expression during development (Wang et al., 2018), dTet was more recently shown to be expressed in a specific glia population, the midline glial cells, where it plays an important role in axon guidance by controlling the expression level of the repulsive cue slit (Ismail et al., 2019). A clear advantage of the *Drosophila* system is the lack of functional redundant TET homologs present in the mammalian system. Therefore, further investigations on the biological role and the catalytic/noncatalytic function of dTet in *Drosophila* may provide valuable insights on the consequences of loss of dTet or ectopic expression of mammalian TETs on gene deregulation during oncogenesis. While the substrate specificity of *Drosophila* Tet is different, demethylating DNA N6-methyladenine (6mA; Zhang et al., 2015; Yao et al., 2018) and RNA 5mC (Delatte et al., 2016), compared with mammalian TETs (for review, see Ismail et al., 2020), its essential function as an important player in neuronal development appears to be conserved from fly to human. Of importance is the more detailed elucidation of the role of human TET3, which shows the highest conservation of structural conformation at its catalytic region compared with dTet (Dunwell et al., 2013). Moreover, ectopic overexpression of TET3 in a glioblastoma cell line was reported to impair cell growth and reduce tumor formation in immunodeficient mouse models (Carella et al., 2020). Intriguingly, increased TET3 expression inhibited growth and self-renewal in tumorigenic glioblastoma stem cells (Cui et al., 2016), indicating that TET3 has tumor suppressor function in the brain. Nevertheless, the impact of overexpressing TET3 *in vivo* has not been studied yet and doing so will shed light on the role of TET3 in development and its correlation with tumorigenesis.

In this study, we first investigated the role of dTet in the *Drosophila* CNS in general and in glial cells in particular. We started by generating a dTet reporter line and found that the dTet reporter is specifically expressed in certain glia populations of the optic lobe called giant glia of the inner and outer optic chiasm. dTet-deficient brains display an overall increase of 6mA levels and an increase in glia cell numbers. Both, cell survival and cell proliferation are deregulated in dTet-deficient brains. This coincides with deregulation of several members of the Hippo signaling pathway that has been implicated in regulation of glia cell proliferation. Furthermore, deficiency of dTet results in midline glia defects in developing brains accompanied by axon commissure defects as well as misdirected and fused Fasciclin 2 (Fas2)-positive axon tracts. These axonal defects were also persistent in adult dTet-deficient brains, where axon guidance defects manifest in two distinct mushroom body phenotypes, a region of the fly brain that is well characterized and associated with olfactory learning and memory (Heisenberg, 2003). Finally, we investigated the role of mammalian TET3 in glial hemostasis by establishing a fly model overexpressing human TET3 in a tissue-specific manner to gain better understanding of the *in vivo* role of TET3. Flies expressing TET3 in dTet expressing tissues displayed significantly reduced lifespan, altered sleep/rest behavior and disturbances in circadian rhythm. Developing brains from this fly population showed a sharp increase in number of glial cells without alterations in proliferation index. In contrast, expression of TET3 in differentiated glia showed only minor defects on the sleep/rest cycle with clear circadian rhythm disturbances in adult flies, but without alterations in glia cell numbers. Hence, our study suggests an important role for dTet in glial cell hemostasis, axon guidance and normal locomotor activity. Furthermore, the data on our human TET3 *Drosophila* model clearly points toward cell-specific effects of TET3 with a more prominent impact on glia in dTet-expressing cells of both developing and adult brains indicating an earlier role of TET3 that supersedes the glial differentiation stage. hTET3 expression in combination with *dTet* knock-down, did show a negative effect on survival and hTET3 was not able to rescue the *dTet* knock-down associated locomotion defects. Further studies on this model may explain the role of TET3 in glia genesis and cancer and shed light on whether the amount of TET3 expressed, and/or cell-specific expression, might be key factors in restoring some of the functional defects observed in dTet-deficient flies.

## Materials and Methods

### Fly husbandry and generation of transgenic fly stocks

All crosses were performed at 25°C and raised on cornmeal-yeast agar under 12/12 h light/dark cycle. The following fly strains were used in this study: wild-type w^1118^, y^1^ w*; Mi[MIC]Tet[MI03920]/TM3, Sb^1^ Ser^1^ (BDSC_44855), Tet[null]/TM6b (Delatte et al., 2016), Exel6091 deficiency w[1118]; Df(3L)Exel6091/TM6B, Tb[1] (BDSC_7570), w1118; UAS-GFP.nls (BDSC_4775) and *repo*-Gal4>UAS-mCD8GFP; *repo*-Gal4/TMb,Tb (gift from M. Hasselblatt), *Tet[MI05009]*-Gal4 (this study), w^1118^;UAS-GFP.nls/CyO; dTet-Gal4/TM6, Tb (this study), Trojan-Gal4 Tet[MI04973] (BDSC_76666), Flag-HA-hTET3/CyO; TM2/TM6TbubiGFP (this study), w[1118]; P[y(+t7.7)w(+mC)=GMR25A01-Gal4]attP2 (BDSC_49102), dTet RNAi (VDRC_102273) and GFP RNAi (gift from M. Hasselblatt).

#### Generation of *dTet*-Gal4 line *Tet[MI05009]*-Gal4

The *Tet[MI05009]*-G4 line was generated through recombinase-mediated cassette exchange of the MiMIC (Minos-mediated integration cassette) insertion site (MI[MIC]TetMI05009; BDSC_38002) located in the 5′ untranslated region of the short *dTet* isoforms and noncoding intronic region of the long *dTet* isoforms with a mutagenic Gal4 gene-trap cassette (containing the *Gal4* coding sequence and *Hsp70* polyadenylation signal) obtained from *Drosophila* Genomics Resource Center (stock #1325) as previously reported (Venken et al., 2011; Extended Data Fig. 2-1*A*,*B*). Four transgenic fly lines were recombinase-mediated cassette exchange had taken place were generated and tested by PCR to determine for orientation of RMCE events within MiMIC insertions. Three of the fly lines had the desired [–] orientation (right-to-left orientation in reference to the chromosome) as has *dTet* and one fly line had [+] orientation (left-to-right orientation in reference to the chromosome). Generation of transgenic flies and orientation confirmation by PCR was conducted by BestGene Inc.

#### Generation of hTET3 transgenic flies

cDNA of human methylcytosine dioxygenase TET3 isoform 1 (sequence ID: NP_001274420.1) was cloned into the fly cloning and transformation vector pUASTattB (Bischof et al., 2007) containing an N-terminal 1xFlag-1xHA tag (MDYKDDDDKLDGGYPYDVPDYAGGLD). Transgenic flies carrying transgene on the third chromosome were generated by BestGene using BDSC_8622 stock.

### Survival and crawling assays

Kaplan–Meier survival curve of wild-type w^1118^ (*n* = 250), Tet[MI03920]/Tet[null] (*n* = 221), Tet[MI03920]/Tet[Df(3L)Exel6091] (*n* = 198) and Tet[null]/Tet[null] (*n* = 227) embryos was generated using GraphPad Prism version 5.01. Flies were staged and collected as 12- to 16-h-old embryos on apple juice plates and their further development examined for a period of 16 d under 12/12 h light/dark cycle at constant temperature (25°C).

For embryonic stage survival assay, 3% agar plates supplemented with fruit juice and sugar were used and yeast paste was added into the middle of the plate. Flies from each genotype were placed inside a cage to mate and lay eggs. To obtain the percentage of lethality at this stage, number of embryos and fraction of hatched embryos was recorded. A chi-square test was used to determine the significance between two groups. Larval survival assays were performed on third instar larvae transferred from culture to new vials, and the number of eclosed adult flies was recorded. Between 80 and 150 larvae were analyzed per group and a chi-square test was used to determine the significance.

Crawling assays were performed in constant light with 50 wandering third instar larvae per group on 15-cm Petri dish containing 2% agarose over graph paper with a 0.2-cm^2^ grid according to previously published work (Nichols et al., 2012). Statistical analysis was performed by ordinary one-way ANOVA and Sidak’s multiple comparison test by comparing control group to either dTet-deficient mutant *p *<* *0.0001 (*n* = 50) using GraphPad Prism version 5.01.

Crawling assays for dTet RNAi knock-down experiments were performed according to previously published work (Fauzi et al., 2020) on 2% agarose plates over graph grid paper under a stereo microscope. Larvae were put into a 1× PBS solution for a few minutes before being transferred to the middle of the plate using a paint brush. The larval distance and the number of larval contractions for 1 min were counted. A total of 50 larvae were collected per genotype and a two-tailed Student’s *t* test was used to determine significance between groups.

Climbing assay was adapted from previously published work (Fauzi et al., 2020). Ten adult flies were transferred to a test tube that was marked at a distance of 6 cm from the bottom. The tube was shaken by hand and the number of flies that could cross the marker line within a time span of 10 s was recorded. The assay was replicated in 6 vials (*n* = 60) and a χ^2^ test was used to determine significance between groups.

### Genomic DNA and RNA isolation from whole larvae, embryos, and larval brain tissues

RNA was extracted from 0.5 to 1 mg 6- to 8-h-old wild-type w^1118^ embryo collection or 80–100 dissected third instar larval brains using the RNeasy Plus Mini kit (#74134) according to manufacturer’s instructions. RNA extraction from whole larvae was performed on 25 wandering third instar larvae washed with 1× PBS using TRIzol (Sigma-Aldrich) according to manufacturer’s instructions. Genomic DNA was extracted from 0.5 to 1 mg 6- to 8-h-old wild-type w^1118^ embryo collection or 80–100 dissected third instar larval brains using DNeasy blood and tissue kit (#69504, QIAGEN) according to manufacturer’s instructions except that DNA samples were subjected to extensive RNase treatments (RNA digestion step was extended from 2 to 30 min).

### Dot blot assay

Fly genomic DNA samples ranging from 100–800 ng were denatured at 95°C for 5 min, directly transferred to ice and spotted on Zeta-Probe GT Genomics tested nylon membranes (#162-0196, Bio-Rad) in 2-μl aliquots. Membranes were prewetted in double distilled dH_2_O and air dried before nucleic acid application. Subsequently, membranes were dried and cross-linked twice at 1200 microjoules [×100] for 50 s with Spectrolinker XL-1000 UV crosslinker. After crosslinking, unbound nucleic acids were removed by washing for 5–10 min in double distilled dH_2_O. Then membranes were blocked in 5% fat-free milk powder in PBS containing 0.1% Tween 20 (PBS-T) for 1 h at room temperature. Next, membranes were incubated with 1:1000 dilution of anti-rabbit 6mA antibody (#202003, Synaptic Systems) overnight at 4°C in 3% milk powder blocking solution. After three rounds of washes with PBS-T, membranes were incubated with 1:5000 dilution of horseradish peroxidase (HRP)-conjugated anti-rabbit IgG (NA934V, GE Healthcare) in blocking solution at 4°C for 2 h. Subsequently, membranes were washed with PBS-T and peroxidase activity was detected with ECL Clarity Max Western Substrate (#1705062, Bio-Rad). Signal detection was done with the ChemiDoc MP system (Bio-Rad). For normalization nucleic acids were stained by 0.4% (w/v) Methylene Blue [Alfa Aesar, Methylene Blue, high purity, biological stain, ThermoFisher (Kandel) GmbH] in equimolar 0.4 m acetic acid: 0.4 m sodium acetate solution (pH 4.7) for 1 h at room temperature.

### 6ma ELISA

6mA ELISA on genomic DNA was performed with 300 ng of genomic DNA as starting material using EpiQuik m6ARNA Methylation Quantification kit (Colorimetric; #P-9005, EpiGentek) according to manufacturer’s instructions with the following modifications: the DNA samples were boiled at 95°C for 5 min and then kept on ice until binding step. A standard curve was performed ranging from 0.02 to 1 ng of 6mA using the positive RNA control provided in this kit. Samples were measured in duplicate or triplicate with a 450-nm filter using plate reader MULTISKAN EX (Thermo Electron corporation).

### Reverse transcription and real-time PCR

RNA was then used to synthesize cDNA using iScript cDNA Synthesis kit (Bio-Rad) or QuantiTec Reverse Transcription kit (#205311, QIAGEN). Real-time PCR was performed using iTaq Universal SYBR Green (Bio-Rad). Reactions were run in duplicates or triplicates on the Bio-Rad CFX-96 Connect. All primers were purchased at Macrogen (Table 1). The ΔΔCT method was used to analyze the relative gene expression normalized to the house-keeping gene *RpL11*. Sample analysis was performed on independent biological duplicates or triplicates. Two-tailed Student’s *t* test was performed to study the statistical significance using GraphPad Prism version 5.01.

### Immunofluorescence staining of larval and adult brains

Third instar larval or adult brains were dissected, fixed in 4% formalin, blocked in 5% normal goat serum (NGS; ab7481, Abcam) 1× PBS (BE17-517Q, Lonza) 0.3% Triton X-100 (TRITON X-100, 0694, Amresco) and subsequently stained using primary antibodies: mouse anti-Repo (1:30, 8D12, DSHB), mouse anti-ELAV (1:20, 9F8A9, DSHB), mouse anti-Fasciclin 2 (1:20, 1D4, DSHB), mouse anti-Fasciclin 3 (1:20, 7G10, DSHB), mouse anti-Robo 1 (1:30, 13C9, DSHB), mouse anti-Slit (1:50, C555.6D, DSHB), mouse anti-Wrapper (1:20, 10D3, DSHB), mouse anti-Bruchpilot (1:20, nc82, DSHB), mouse anti-Prospero (1:100, MR1A, DSHB), rabbit anti-Asense (Ase; 1:200, ab117839, Abcam), Alexa Fluor 488-conjugated rabbit anti-HRP (1:300, 323-545-021, Jackson ImmunoResearch), rabbit anti-Phospho(Ser10)-H3 (1:500, Millipore), cleaved *Drosophila* Dcp-1 (Asp216) antibody (1:200, #9578, Cell Signaling Technology), CyCE-s (8B10) 1:10 (kind gift from H. Richardson (Richardson et al., 1995) and anti-rabbit m6A 1:300 (#202003, Synaptic Systems), anti-HA (16B12, Biolegend). As secondary antibodies goat anti-mouse IgG H&L Alexa Fluor 488 (1:500, ab150117, Abcam), goat anti-mouse IgG H&L Alexa Fluor 594 (1:500, ab150116, Abcam), goat anti-rabbit IgG H&L Alexa Fluor 488 (1:500, ab150077, Abcam), goat Alexa Fluor 594 anti-rabbit IgG H&L (1:500, ab150080, Abcam), goat anti-rabbit Alexa Fluor 635 (1:500, A31576, Life Technologies) were used. As a nuclear counterstain 4′,6′-diamidino-2-phenylindole dihydrchloride (DAPI; 1:5000, #D1306, Invitrogen) was used. Brains were mounted in ProLong Gold antifade reagent (REF-P36930, Invitrogen).

### Image acquisition, analysis, and quantification

For image acquisition of larval and adult brains Zeiss LSM 710 laser scanning confocal microscope (Biological Imaging Core Facility, American University of Beirut) was used. Zen 2009 software (Zeiss), ImageJ software (available at http://imagej.nih.gov/ij/), and Fiji (Schindelin et al., 2012) were employed for image processing. For quantification of pS10-H3-positive cells the ImageJ plugin Image-based Tool for Counting Nuclei (ITCN) was used. Unpaired *t* test was performed to determine statistical significance of increase or decrease in sample groups compared with control groups using GraphPad Prism version 5.01.

### Quantification and scoring of different brain phenotypes

For quantification of brain phenotypes including midline glia defects, commissure defects, and mushroom body defects, visual image scoring was conducted blindly using the Software Blinder (Cothren et al., 2018) and the following scoring systems:

1. Scoring system midline phenotype: 0, no phenotype; 1, one to two midline segments disorganized or misoriented; 2, more than two midline segments disorganized or misoriented. The grading system was developed according to the severity of the midline phenotypes observed (number of midline segments disorganized or misoriented).
2. Scoring system commissure phenotype: 0, no abnormal commissures; 1, single abnormal commissure; 2, two or more abnormal commissures. The grading system was adapted from previously published work (Mosca and Schwarz, 2010).
3. Scoring system mushroom body lobe phenotype: no phenotype; misdirected or missing α-lobe(s); misdirected or missing β-lobe(s); misdirected or missing α- and β-lobe(s). The grading system was adapted from previously published work (Michel et al., 2004).
4. Scoring system mushroom body midline crossing: 0, no phenotype; 1, mild fusion; 2, moderate fusion, 3, complete fusion. The grading system applied is published previously (Kelly et al., 2017).

### Monitoring locomotion activity of adult flies using *Drosophila* activity monitor (DAM)

The DAM system enables to acquire and compare activity data of different genotypes with a large number of flies. A total of 32 male flies were placed in one DAM each into an individual channel equipped with an infrared light beam to detect movement when interrupted. Flies were kept under a 12/12 h light/dark cycle at constant temperature (25°C) and constant humidity (80%) on sucrose (5%)/agar (1%) media in glass tubes sealed with paraffin to avoid dehydration. Considering 5 min of inactivity as sleep and >24 h of immobility as a death event, the obtained data can be processed to quantify locomotor activity, sleep duration and survival of the flies. Trikinetics data acquisition software (DAMSystem308, Trikinetics Inc.) saves activity data as channel counts per time period. ImageJ software (available at http://imagej.nih.gov/ij/) combined with the freely available ActogramJ plug-in (v0.9; Schmid et al., 2011) was used to draw periodic actograms for each individual fly. Calculation and statistical evaluation of raw data were conducted using Microsoft Excel and GraphPad Prism (GraphPad Software). All analyzed activity and sleep parameters were calculated for each day of the experiment as average from data of all living flies at this time and afterward displayed over the duration of the experiment (up to 20 d).

### Graphs and statistical analysis

All graphs were generated and statistical analysis performed using GraphPad Prism version 5.01.

## Results

### dTet is required for *Drosophila* development and flies with dTet deficiency have severe locomotion defects

**Figure 1.**
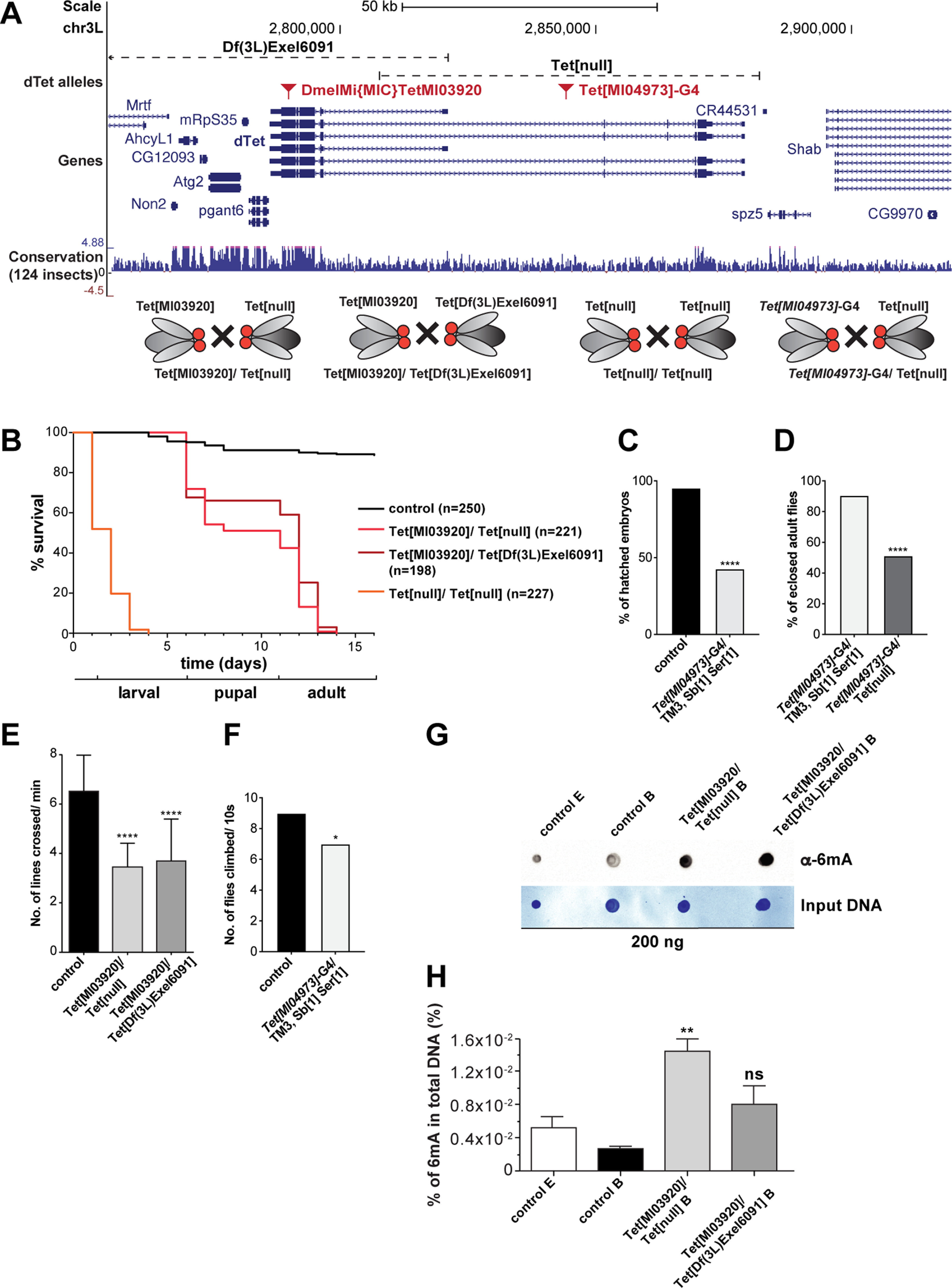
dTet is required for *Drosophila* development and dTet-deficient flies have a severe locomotion phenotype. ***A***, Schematic showing the cytogenetic location of Tet[MI03920] and *Tet[MI04973]*-G4 insertion, Tet[Df(3L)Excel6091] deficiency and Tet[null] deletion on *Drosophila* Chromosome 3 that were used to generate dTet-deficient flies. In order to generate dTet-deficient animals, Tet[MI03920] was either crossed to Tet[null] or to Tet[Df(3L)Excel6091]. In addition, Tet[null] was crossed to itself or to Tet[MI04973]. ***B***, Kaplan–Meier survival curve of wild-type (*n* = 250), Tet[MI03920]/Tet[null] (*n* = 221), Tet[MI03920]/Tet[Df(3L)Excel6091] (*n* = 198), and Tet[null]/Tet[null] (*n* = 227) embryos is shown. Note that only 40–60% of dTet-deficient flies survived to the adult stage and those died within 2 d of eclosion. All Tet[null]/Tet[null] animals died as embryos or in early larval stages. ***C***, Embryo survival assay of wild-type embryos (*n* = 218) compared with Tet[MI04973]-G4/TM3, Sb[1] Ser[1] heterozygous embryos (*n* = 159). The group of Tet[MI04973]-G4/TM3, Sb[1] Ser[1] heterozygous embryos showed an early lethality at the embryonic stage in >50% of observed animals. A chi-square test was used to determine the significance between the two groups (****p < 0.0001). ***D***, Larval survival assay comparing Tet[MI04973]-G4/TM3, Sb[1] Ser[1] animals (*n* = 150) to Tet[MI04973]-G4/Tet[null] (*n* = 80) animals showed that only 50% of Tet[MI04973]-G4/Tet[null] animals eclose to adults, significantly less than in the control group. Statistical analysis was performed by a chi-square test (****p < 0.0001). ***E***, Crawling assays with wandering third instar larvae on 0.4-cm grid paper showed that Tet[MI03920]/Tet[null] (*n* = 50) and Tet[MI03920]/Tet[Df(3L)Excel6091] animals (*n* = 50) have defective crawling activity. Statistical analysis was performed by ordinary one-way ANOVA and Sidak’s multiple comparison test (****p <* *0.0001). At adult stages, both mutants are incapable to fly or climb. ***F***, Climbing assay displaying the average number of adults that were able to reach a 6-cm mark within 10 s (*n* = 60). Heterozygous *Tet[MI04973]*-G4/TM3, Sb[1] Ser[1] flies showed significantly reduced mobility compared with wild-type flies. Statistical analysis was performed by a chi-square test (*p = 0.0107). ***G***, Dot blot assay was performed on 200 ng of indicated genomic DNA samples using an anti-6mA antibody (up). Methylene blue staining was performed as DNA input control (down). ***H***, Quantification of 6mA levels in total genomic DNA (300 ng) using a commercial m6A ELISA kit. Statistical analysis was performed by ordinary one-way ANOVA and Bonferroni’s multiple comparison test. [**p <* *0.01 (*n* = 3); ns, *p *>* *0.05 (*n* = 2)]. Genomic DNA samples are as follows: control E: 6- to 8-h-old wild-type embryos; control B: brains from wild-type third instar wandering larvae; Tet[MI03920]/Tet[null] B: brains from Tet[MI03920]/Tet[null] third instar wandering larvae and Tet[MI03920]/Tet[Df(3L)Excel6091] B: brains from Tet[MI03920]/Tet[Df(3L)Excel6091] third instar wandering larvae. See also Extended Data Figure 1-1.

To investigate the role of dTet in the fly brain, we generated dTet-deficient flies by crossing Tet[MI03920] (DmelMI[MIC]TetMI03920) allele to either Tet[null] allele (Delatte et al., 2016; Tet[MI03920]/Tet[null]) or to Tet[Exel6091] (Df(3L)Exel6091) allele (Tet[MI03920]/Tet[Exel6091]). In addition, Tet[null] was crossed to itself and homozygous Tet[null] embryos were collected (Tet[null]/Tet[null]). Moreover, Tet[MI04973]-G4 flies, a Trojan-Gal4 line in which the Mi[MIC]MI04973 insertion has been replaced with a gene trap cassette containing a Trojan-Gal4 exon as well as an Hsp70 transcription termination signal, were either used heterozygous or crossed to Tet[null]. Mi[MIC]MI04973 is located in a coding intron of the four long *dTet* isoforms (Fig. 1*A*; Extended Data Fig. 1-1*A*). Because of lack of publicly available dTet antibody, we could not test for absence of dTet protein in the four different dTet mutants, but we tested three of the four mutants for levels of *dTet* mRNA expression as well as expression of directly adjacent genes using quantitative PCR (Extended Data Fig. 1-1*B*,*D*). While Tet[null]/Tet[null] embryos showed complete absence of *dTet* mRNA, combinations with the Tet[MI03920] allele showed about half the amount of *dTet* mRNA expression compared with wild-type. This is expected, since the MiMIC insertion contains stop codons in all three reading frames resulting in premature translation termination in the last coding exon of all six dTet isoforms without affecting transcription (Fig. 1*A*; Extended Data Fig. 1-1*A–D*). Survival rates of a wild-type control compared with different dTet-deficient allelic combinations showed that about half of Tet[MI03920]/Tet[null] and Tet[MI03920]/Tet[Exel6091] animals die during the pupal stage with 40–60% of flies eclosing. As reported previously (Zhang et al., 2015), none of the eclosed dTet-deficient flies survived more than 3 d posteclosion. Tet[null]/Tet[null] flies died as embryos or in early larval stages, and thus were not used for any additional experiments, since we focused our study on larval and adult stages (Fig. 1*B*). Tet[MI04973]-G4/TM3, Sb[1] Ser[1] heterozygous animals showed an early embryonic lethality (Fig. 1*C*). Tet[MI04973]-G4/Tet[MI04973]-G4 homozygous animals all died as embryos or in early larval stages (data not shown). Tet[MI04973]-G4/Tet[null] heterozygous animals showed a similar survival phenotype to the other Tet allele combinations with ∼50% of adults eclosing (Fig. 1*D*) that survived maximal 2 d posteclosion. The differences between survival rates in homozygous and heterozygous dTet allele combinations could either be because of a second (off-target) mutation on the Tet alleles, which make the homozygous alleles less fit, or can indicate only partial loss of function of some of the Tet mutant alleles. Interestingly, Tet[MI03920]/Tet[null] and Tet[MI03920]/Tet[Exel6091] third instar larvae, both displayed highly significant reduction in locomotion compared with wild-type control larvae as shown by crawling assays (Fig. 1*E*). This is in line with previously published knock-down experiments of dTet reporting locomotion defects (Wang et al., 2018; Ismail et al., 2019). Adult dTet-deficient flies cannot fly or climb and are prone to fall over while walking. In fact, even Tet[MI04973]-G4/TM3, Sb[1] Ser[1] animals that still have one dTet wild-type allele displayed impaired climbing activity compared with wild-type flies (Fig. 1*F*). Since dTet was reported to demethylate 6mA in genomic DNA (Yao et al., 2018; Zhang et al., 2015), we decided to measure the levels of 6mA in genomic DNA from wild-type versus dTet-deficient fly brains using dot blot and 6mA ELISA assay. As an additional control, we used genomic DNA from 6–8 h old wild-type embryos. With both assays, 6mA levels were higher in dTet-deficient brains compared with wild-type brains or embryos, further validating the function of dTet as a 6mA demethylase in fly brains (Fig. 1*G*,*H*).

10.1523/ENEURO.0418-21.2022.f1-1 Extended Data Figure 1-1 Changes in expression of dTet and neighboring genes in dTet-deficient flies. ***A***, Gene browser tracks showing the cytogenetic location of Tet[MI03920] insertion, Tet[Df(3L)Excel6091] deficiency, and dTet[null] deletion on *Drosophila* Chromosome 3 that were used to generate *dTet*-deficient flies. Note that Tet[Df(3L)Excel6091] deficiency lacks the C-terminal part of *dTet* gene including the well-conserved catalytical domain as well as several additional genes upstream of *dTet* including *pgant6*, *CG12093*, *AhcL1*, *Mrtf*, *Fife*, etc. Tet[MI03920] fly strain has a MiMIC transposon containing a gene-trap cassette and the yellow^+^ marker flanked by two inverted bacteriophage ΦC31 integrase attP sites in the last exon of *dTet* thus interrupting the catalytic domain of all six *dTet* isoforms. In order to generate *dTet*-deficient animals, Tet[MI03920] flies were either crossed to Tet[null] deletion (Tet[MI03920]/Tet[null]) or to Tet[Df(3L)Excel6091] deficiency (Tet[MI03920]/Tet[Df(3L)Excel6091]). ***B***, Relative expression of *dTet* and several adjacent genes including *Fife*, Mrtf, *AhcL1*, *CG12093*, and *spz-5* in Tet[MI03920]/Tet[Df(3L)Excel6091] flies. Note that all genes missing in Tet[Df(3L)Excel6091] deficiency show about 50% reduction in expression levels. *dTet* also showed only 50% reduction in expression level, indicating that Tet[MI03920] insertion allele can produce *dTet* mRNA. ***C***, Relative expression of *dTet* and directly upstream located gene *spz-5* in Tet[MI03920]/Tet[null] and (***D***) Tet[null]/Tet[null] flies. Expression level of *spz-5* is not affected in Tet[MI03920]/Tet[null] or Tet[null]/Tet[null] flies. Tet[MI03920]/Tet[null] flies showed only reduced levels of *dTet* mRNA due to the Tet[MI03920] insertion allele, while no *dTet* mRNA was detected in Tet[null]/Tet[null] flies. Genotypes are as follows: control: wild-type w^1118^; Tet[MI03920]/Tet[null]; Tet[MI03920]/Tet[Df(3L)Excel6091] and Tet[null]/Tet[null]. Displayed values are mean of two biological replicates (*n* = 2, 25 larvae per group) each averaged from two to three technical replicates shown with SEM normalized to the house-keeping gene *RpL11*. Graphs and statistical analysis by unpaired Student’s *t* test were generated using GraphPad Prism version 5.01 (****p <* *0.0001, ***p <* *0.001, **p <* *0.01, *p <* *0.05) and (ns, *p *>* *0.05). Download Figure 1-1, TIF file.

### dTet is expressed in most neurons and in specific glia populations in the optic lobe

**Figure 2.**
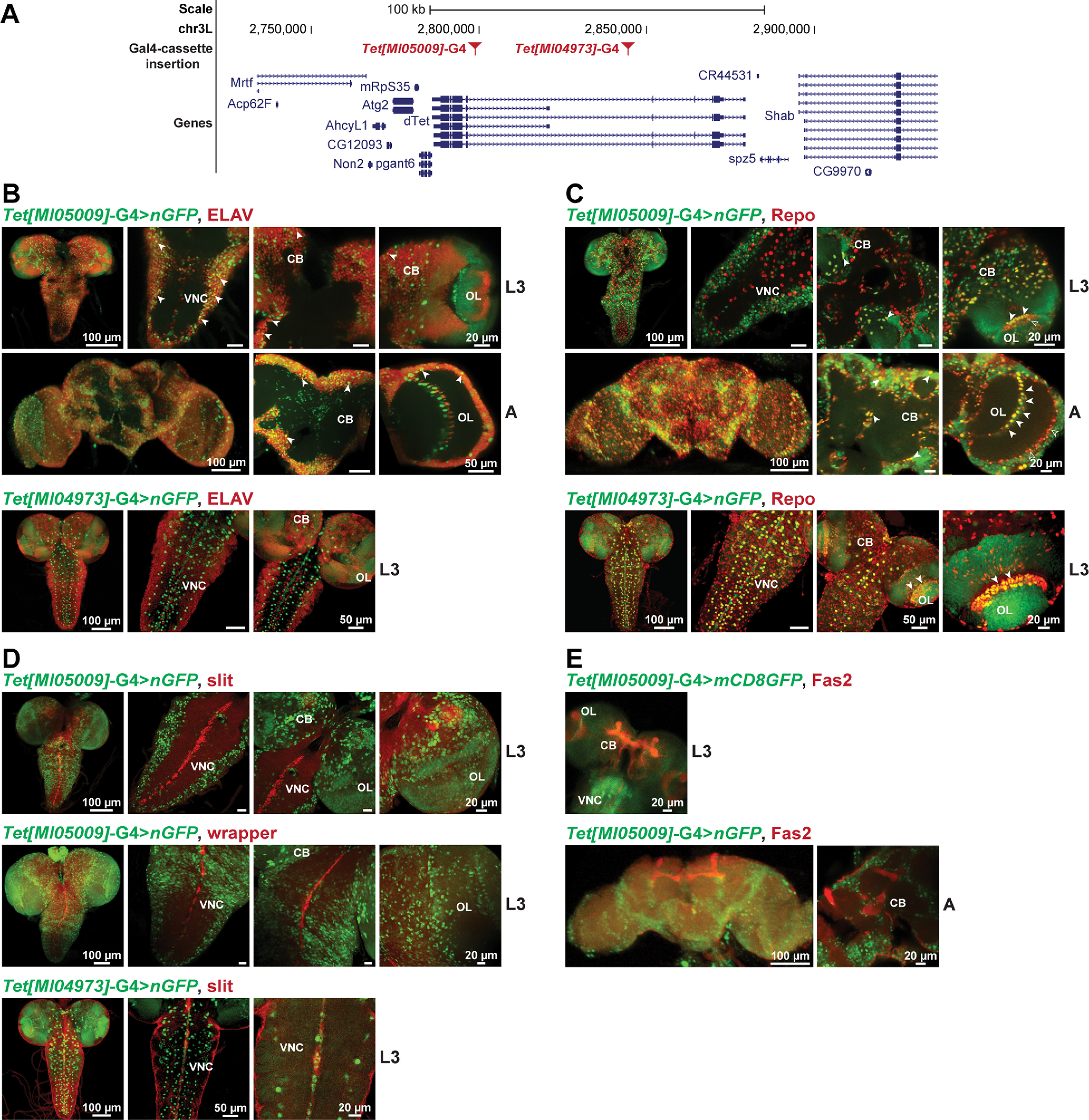
dTet is expressed in most neurons and in specific glia populations in the optic lobe of larval and adult brains. Genomic location of Gal4 cassette insertion site of *Tet[MI05009]*-G4 and *Tet[MI04973]*-G4 (***A***). Immunofluorescence co-staining of whole-mount *Tet[MI05009]*-G4> UAS-nuclear GFP/UAS-mCD8GFP or *Tet[MI04973]*-G4.UAS-nuclear GFP larval (L3) or 1-d-old adult (***A***) brains with five different markers including (***B***) anti-ELAV (neuronal cell marker), (***C***) anti-Repo (glial cell marker), (***D***) anti-slit (expressed in ventral midline, mushroom body, and others) and anti-wrapper (midline glia marker) as well as (***E***) anti-Fas2 antibody marking the mushroom body, longitudinal fascicles of the VNC and a subset of neurons. White arrowheads mark cells expressing both, dTet and the designated brain marker. See also Extended Data Figure 2-1. VNC: ventral nerve cord, CB: central brain, OL: optic lobe.

To characterize which cells in the CNS show dTet reporter expression, we generated a *dTet*-Gal4 line, *Tet[MI05009]*-G4 (see Materials and Methods, Fly husbandry and generation of transgenic fly stocks; Fig. 2*A*, Extended Data Fig. 2-1*A*,*B*) and crossed it with either a nuclear or membrane targeted GFP reporter, nGFP and mCD8GFP, respectively. Moreover, we used a second, publicly available Trojan-Gal4 line, *Tet[MI04973]*-G4 to compare and validate expression patterns. Subsequently, we dissected third instar larvae and adult brains from *Tet[MI05009]*-G4>UAS-GFP animals as well as third instar larvae brains from *Tet[MI04973]-*G4>UAS-GFP animals and stained them with a range of neuronal and glia cell markers. Co-staining with the pan-neuronal marker Embryonic lethal abnormal vision (ELAV) showed that *Tet[MI05009]*-G4 is expressed in most neurons of the ventral nerve cord (VNC; L3) and adult central brain (Fig. 2*B*) similar what has been published for an endogenous *dTet*-GFP reporter (Ismail et al., 2019). *Tet[MI04973]-*G4 on the other side, showed less overlap with the neuronal marker ELAV (Fig. 2*B*). Co-staining with the homeodomain transcription factor Reversed polarity (Repo) marking all glial cells, except midline glia, revealed that while *Tet[MI05009]*-G4 is expressed in a considerable number of glial cells in the central brain, only few glial cells of the VNC display *Tet[MI05009]*-G4 expression (Fig. 2*B*). Interestingly, *Tet[MI05009]*-G4 is specifically expressed in a certain glia population of the optic lobe called giant glia of the inner optic chiasm (IOC; Fig. 2*C*; Extended Data Fig. 2-1*C*, white arrowheads) and in few cells that are part of the giant glia of the outer chiasm (Fig. 2*C*, empty arrowheads) from L3 onwards. Consistently, *Tet[MI04973]-*G4 is also expressed in a considerable number of glial cells in the central brain and in giant glia of the IOC. Contrary to *Tet[MI05009]*-G4, it is also expressed in many glial cells of the VNC (Fig. 2*C*). In fact, most of the *Tet[MI04973]-*G4 expression overlaps with the glial cell marker Repo. Studies have shown that chiasm glia are born early in larval life and thus may participate in the development of the optic lobe (Tix et al., 1997). During pupal development, optic chiasm glia gradually arrange into two rows: the outer chiasm glia are positioned in the periphery (Fig. 2*C*, lower panel, empty arrowheads) and the inner chiasm glia are found in the middle of the optic lobe (Fig. 2*C*, lower panel, white arrowheads). Each chiasm contains one stack of giant glia, enwrapping and enclosing a high density of axons (Extended Data Fig. 2-1*C*). This strategic location at fiber crossings implies that giant glia might be involved in neuronal pattern formation and provide cues for outgrowing axons (Tix et al., 1997). Overlapping expression in the midline glia (L3) was not observed when we co-stained *Tet[MI05009]*-G4>UAS-nGFP larvae with either of the two midline glia markers, slit or wrapper (Fig. 2*D*), but was observed with *Tet[MI04973]-*G4>UAS-nGFP larvae stained with slit. Besides, midline glia expression of dTet has previously been reported using an endogenous dTet-reporter (Ismail et al., 2019). Moreover, there was no overlap detected between Fas2 and dTet reporter expression in adult fly brains, whereas in larval stages, *Tet[MI05009]*-G4>mCD8GFP brains showed a faint signal in the mushroom body region (Fig. 2*E*). To summarize, three different dTet reporters exhibit clear variations in expression patterns possibly because of their different locations within the dTet locus. However, the expression of dTet in specific glia populations is further verified with expression of dTet in optic chiasm glia observed with three independent reporters and expression of dTet in midline glia observed with two reporters.

10.1523/ENEURO.0418-21.2022.f2-1 Extended Data Figure 2-1 Generation of *Tet[MI05009]*-G4 line. ***A***, Genomic location of Gal4 cassette insertion site MI[MIC]TetMI05009 located in the 5′ untranslated region of the short dTet isoforms. MiMIC consists of two *Minos* inverted repeats (MiL and MiR), two inverted ΦC31 integrase *attP* sites, a gene-trap cassette consisting of a splice acceptor site (SA) followed by stop codons in all three reading frames and the *EGFP* coding sequence with a polyadenylation signal (*pA*), and the yellow^+^ marker. The sequence between the *attP* sites was replaced via RMCE with a plasmid containing a Gal4 sequence between two inverted *attB* sites, resulting in insertion of the Gal4 sequence between *attL* and *attR* sites. Recombination can occur in reverse or forward orientation relative to the targeted locus and is shown here in the forward orientation. ***B***, Expression of UAS-GFP using *Tet[MI05009]*-G4 line results in green fluorescent protein expression in all dTet-expressing cells. ***C***, Immunofluorescence co-staining of whole-mount *Tet[MI05009]*-G4> UAS-mCD8 GFP larval (L3) brains that express membrane targeted green fluorescent protein through the *Tet[MI05009]*-G4 driver with anti-Repo (glial cell marker). RMCE, recombinase-mediated cassette exchange; MiMIC, Minos-mediated integration cassette. Download Figure 2-1, TIF file.

### dTet-deficient larvae display distinct brain phenotypes

**Figure 3.**
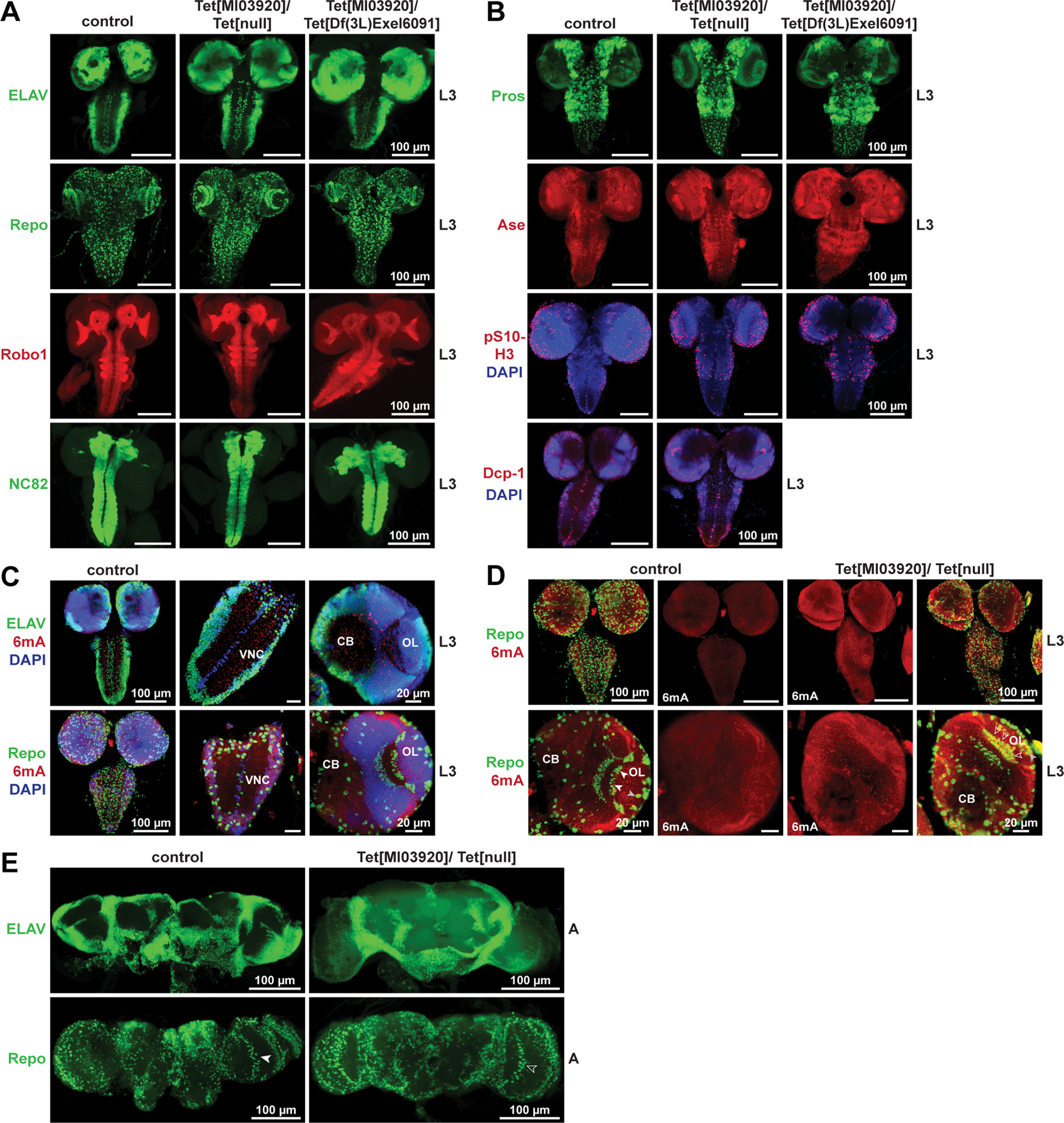
dTet-deficient larvae display distinct brain phenotypes. Whole-mount control (wild-type w*^1118^*), Tet[MI03920]/Tet[null] and Tet[MI03920]/Tet[Df(3L)Excel6091] third instar larval brains were dissected fixed and stained with (***A***) anti-ELAV (neuronal cell marker), anti-Repo (glial cell marker), anti-Robo1 (neuropile marker) and anti-NC82 (synaptic/active neuropile marker), (***B***) two neural progenitor markers, anti-Prospero (controls neuronal identity in a subset of neuroblast progeny and initiates the development of GMCs) and anti-Ase (marks embryonic/larval neuroblasts) as well as anti-phospho-Ser10 histone H3 (pS10-H3, mitotic marker) and anti-cleaved Death caspase-1 (Dcp-1, apoptosis marker). ***C***, Whole-mount control (wild-type w^1118^) brains were dissected fixed and stained with ELAV and 6mA (epigenetic DNA mark, possible dTet substrate) or Repo and 6mA to determine a rough 6mA profile for larval brains. ***D***, Whole-mount control and Tet[MI03920]/Tet[null] third instar larval brains were dissected fixed and stained with anti-Repo and 6mA. In Tet[MI03920]/Tet[null] brains glial cells of the IOC display slightly increased 6mA signal (open arrowheads versus white arrowheads). Contrary, several glia in the periphery show comparable 6mA signal in control and Tet[MI03920]/Tet[null] brains (open arrowhead). ***E***, Whole-mount control and Tet[MI03920]/Tet[null] adult brains were dissected fixed and stained with anti-ELAV or anti-Repo. In Repo staining, arrowheads mark the giant glia of the IOC. In dTet-deficient brains this glia population appeared scattered (open arrowhead) and not arranged in a row in the middle of the optic lobe as observed for control brains (white arrowhead). Note that maximum z-projections are displayed for all markers.

In order to further investigate the role of dTet in the brain, we dissected Tet[MI03920]/Tet[null] and Tet[MI03920]/Tet[Df(3L)Exel6091] brains of third instar larvae and compared them to wild-type control brains by immunofluorescence staining using a panel of markers including Repo, ELAV, and Robo-1, the later marking the neuropil region, as well as NC82, labeling Bruchpilot, a component of the presynaptic active zone that is essential for structural integrity and function of synaptic active zones (Wagh et al., 2006). Notably, panneuronal RNAi knock-down of Bruchpilot leads to locomotor inactivity and instable flight in flies (Wagh et al., 2006), a phenotype similar to what is observed for dTet-deficient flies. However, no obvious defects in Tet[MI03920]/Tet[null] or Tet[MI03920]/Tet[Df(3L)Exel6091] brains were detected (Fig. 3*A*). Furthermore, we examined markers for progenitor cell populations including homeodomain transcription factor Prospero (Pros) that is expressed in nuclei of ganglion mother cells (GMCs) as well as in undifferentiated neurons and Ase that is expressed in type 1 larval neuroblasts. There was no clear difference visible between control and Tet[MI03920]/Tet[null] and Tet[MI03920]/Tet[Df(3L)Exel6091] brains (Fig. 3*B*). We then assessed occurrence and distribution of cell proliferation and apoptosis, two important processes during development in dTet-deficient brains. For this, we checked cell proliferation by staining for phospho(Ser10)-H3, which detects mitotically active cells. As expected, the pS10-H3 signal was particularly strong in the outer and inner proliferation center of the optic lobes and the upper VNC. At low magnification, we observed more cell proliferation in Tet[MI03920]/Tet[null] and Tet[MI03920]/Tet[Df(3L)Exel6091] mutants compared with control brains (Fig. 3*B*). Staining for cleaved Death caspase-1 (Dcp-1) did not show any major differences for Tet[MI03920]/Tet[null] brains compared with control brains (Fig. 3*B*, lower panel) suggesting similar rates of apoptosis. Since 6mA in DNA was reported as the major substrate of dTet in flies (Zhang et al., 2015; Yao et al., 2018), we subsequently stained to detect 6mA levels in neurons and glia cells (Fig. 3*C*) and investigated how 6mA levels in glia cells are affected in Tet[MI03920]/Tet[null] brains compared with control brains. As seen by more quantitative assays including dot blot and ELISA (Fig. 1*G*,*H*), we detected an increase of 6mA signal in Tet[MI03920]/Tet[null] brains. Higher magnification images of the optic lobes showed that giant glia of the IOC and OC that specifically express dTet reporter (Fig. 2*C*, white arrowheads) possibly show lower levels of 6mA in control brains (Fig. 3*D*, white arrowheads) than in Tet[MI03920]/Tet[null] brains (Fig. 3*D*, empty arrowheads). Contrary, some glia cells in the periphery of the optic lobes that do not express dTet display more comparable 6mA signal in both, control and Tet[MI03920]/Tet[null] brains (Fig. 3*D*, gray arrowheads). Last, we checked whether Tet[MI03920]/Tet[null] brains of 1-d-old adults displayed any defects in neuron or glia populations compared with age matched control flies. We found that the glia population in the optic lobe between medulla and lobula that shows *Tet[MI05009]*-G4>nGFP expression (Fig. 2*C*, second panel) formed a straight line in control brains (white arrowhead), but appeared less organized in Tet[MI03920]/Tet[null] brains (Fig. 3*E*, open arrowhead).

### dTet-deficient brains display clear defects in midline glia organization and axon guidance

**Figure 4.**
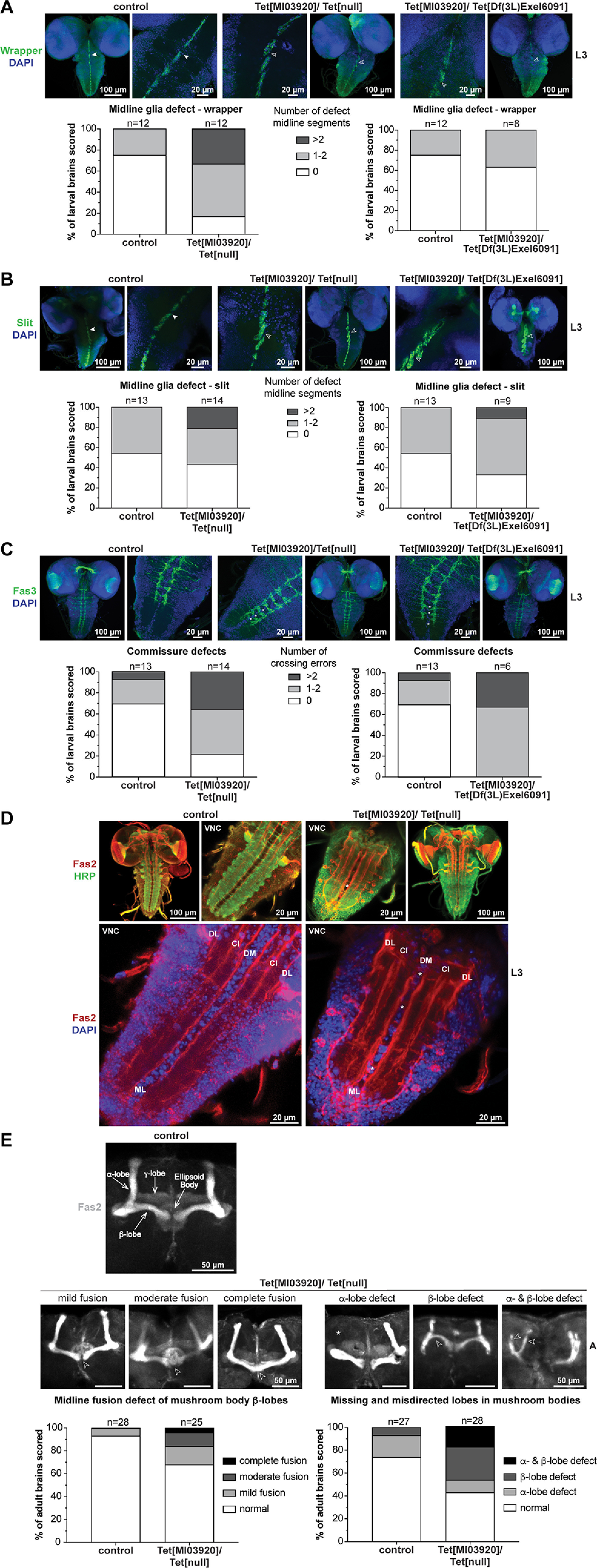
dTet-deficient larvae display clear defects in midline glia organization and axon guidance. Whole-mount control (wild-type w*^1118^*), Tet[MI03920]/Tet[null] and Tet[MI03920]/Tet[Df(3L)Excel6091] larval brains were dissected fixed and stained with (***A***) anti-wrapper (midline glial cell marker). Higher magnification images (63× objective) of midline glia marked with wrapper showed that the organization of midline glia is disrupted in Tet[MI03920]/Tet[null] and Tet[MI03920]/Tet[Df(3L)Excel6091] larval brains (open arrowheads) compared with control brains (white arrowheads). For quantification of observed midline glia defects wrapper stains were scored blindly according to the indicated scoring system, where *n* corresponds to number of brains scored per group with control (*n* = 12), Tet[MI03920]/Tet[null] (*n* = 12) and Tet[MI03920]/Tet[Df(3L)Excel6091] (*n* = 8). ***B***, Anti-slit (expressed in ventral midline and mushroom body among others). Higher magnification images (63× objective) of midline glia marked with slit showed that the organization of midline glia is disrupted in Tet[MI03920]/Tet[null] and Tet[MI03920]/Tet[Df(3L)Excel6091] larval brains (open arrowheads) compared with control brains (white arrowheads). For quantification of midline glia defects slit stains were scored blindly according to the indicated scoring system, where *n* corresponds to number of brains scored per group with control (*n* = 13), Tet[MI03920]/Tet[null] (*n* = 14) and Tet[MI03920]/Tet[Df(3L)Excel6091] (*n* = 9). ***C***, Whole-mount control, Tet[MI03920]/Tet[null] and Tet[MI03920]/Tet[Df(3L)Excel6091] larval brains were dissected fixed and stained with anti-Fas3 (labeling one of the lateral axon tracts running lengthwise through the nerve cord and a band of axons that cross the midline in each segment). High-magnification images (63× objective) revealed that commissures crossing the midline appeared mostly discontinuous as if broken at the center (white stars) in the lower part of the VNC with some aberrant crossings in the upper part of the VNC (empty arrowhead). The graph below displays blinded scoring of disrupted horizontal commissure crossings according to the indicated scoring system, where n corresponds to number of brains scored per group with control (*n* = 13), Tet[MI03920]/Tet[null] (*n* = 14) and Tet[MI03920]/Tet[Df(3L)Excel6091] (*n* = 6). ***D***, Whole-mount control and Tet[MI03920]/Tet[null] larval brains were dissected fixed and co-stained with anti-Fas2 antibody marking a subset of VNC axons and HRP (staining all VNC axons). In high-magnification images (63× objective, lower panel), Fas2 longitudinal tracts are designated by letters relative to their position in the dorsoventral (D, dorsal; C, central) and mediolateral (M, medial; I, intermediate; L, lateral) position. Cells of the midline (ML) are clearly visible with DAPI stain. Fas2-positive axons that cross the midline (ML) in Tet[MI03920]/Tet[null] mutants are marked by white stars. ***E***, One-day-old adult control and Tet[MI03920]/Tet[null] *Drosophila* brains were dissected, fixed, and visualized using anti-Fas2 antibody that stains mushroom body axons. As seen in the control the mushroom body cells extend several axons bundles (so-called lobes) including dorsally projecting α-lobes, medially-projecting β- and γ-lobes. The expression of Fas2 in γ-lobes is weaker than in α- and β-lobes. The centrally located ellipsoid body is also visualized by Fas2 staining. Importantly, β-lobes of control flies usually terminate before the brain midline. The mushroom body axons of Tet[MI03920]/Tet[null] brains displayed multiple phenotypes including varying amounts of β-lobe mis-projection (open arrowheads) across the midline that was rarely observed in control brains (lower panel, left side) as well as frequently missing (white asterisk) or misdirected (open arrowheads) α- and/or β-lobes (lower panel, right side). Quantification of mushroom body phenotypes was done according to the indicated scoring systems. Maximum intensity z-stack projections of representative examples of each scored category are displayed.

dTet was recently reported to be expressed in midline glia (Ismail et al., 2019), a highly-specialized cell type that is molecularly, functionally and developmentally distinct from other glia and corresponds to a structure in vertebrates called the floor plate (Jacobs, 2000). Here, we confirmed midline glia expression with another dTet reporter (Fig. 2*D*) and analyzed whether the midline glia organization is affected in dTet-deficient brains by staining for Slit protein, a repulsive signal secreted by midline glia (Battye et al., 1999), and the midline glia marker Wrapper (Noordermeer et al., 1998). We found that dTet-deficient brains exhibit defects in the midline glia structure characterized by at least one of the midline segments being disoriented or misaligned (Tet[MI03920]/Tet[null], empty arrowheads) in the normally straight midline (control, white arrowheads) for both analyzed markers (Fig. 4*A*,*B*), suggesting a disrupted migration of midline glia in the absence of dTet. Quantification of this midline phenotype showed that 60–80% of dTet-deficient brains display defects in the midline glia with 20–30% displaying more severe defects (more than two midline segments affected; Fig. 4*A*,*B*).

Since midline glia play an important role in axon guidance by controlling repulsive and attractive signals during development, we wanted to investigate whether axon connections are affected in dTet-deficient mutants. Therefore, staining with Fasciclin 3 antibodies (Fas3) labeling one of the lateral axon tracts running lengthwise through the nerve cord and a band of axons that cross the midline in each segment, was conducted (Fig. 4*C*). As reported previously for *tubulin* and *sim*-Gal4-driven RNAi-mediated dTet knock-down (Ismail et al., 2019), dTet-deficient mutants displayed disruptions in axons and midline commissure organization in the VNC (Fig. 4*C*). The midline crossings appeared mostly discontinuous as if broken at the center (white stars) in the lower part of the VNC with some aberrant crossings in the upper part of the VNC (empty arrowhead). Blinded quantification of disrupted horizontal commissure crossings in larval brains detected at least one error in >80% of Tet[MI03920]/Tet[null] brains with >30% exhibiting two or more errors (Fig. 4*C*). Similar results were obtained for Tet[MI03920]/Tet[Df(3L)Exel6091] brains, where 100% of brains displayed at least one error and >30% showed more severe defects with two or more crossing errors (Fig. 4*C*). Next, co-staining with Fasciclin 2 antibody (Fas2) and HRP was conducted. Fas2 marks a subset of VNC axons including the longitudinal fascicles of the VNC that serve as a set of evenly distributed landmarks, since they remain comparatively constant between specimens and over developmental time (Landgraf et al., 2003). HRP, on the other side, stains all VNC axons. Fas2-positive axon pathways in Tet[MI03920]/Tet[null] brains were less organized compared with control, especially the longitudinal axons in the lower VNC are closer together and look more clustered as if they are fused together (Fig. 4*D*). In fact, it looked as if the midline (ML) of Tet[MI03920]/Tet[null] brains contains less cells compared with control brains when comparing the DAPI stains. Additionally, few Fas2-positive axons cross the midline (ML) in Tet[MI03920]/Tet[null] mutants (white stars). These midline crosses are completely absent in control brains. Subsequently, we analyzed the mushroom body morphology of 1-d-old adults by Fas2 antibody staining. Mushroom bodies are highly plastic brain regions essential for many forms of learning and memory (Heisenberg, 2003). Accordingly, defects in axonal guidance proteins often cause incompletely penetrant mushroom body phenotypes (Michel et al., 2004; Ng, 2012; Kelly et al., 2016). Fas2 is enriched on α- and β-axon branches and to a lesser extent on the γ-lobes and regions of the ellipsoid body (Fig. 4*E*). We identified two distinct mushroom body defects in adult Tet[MI03920]/Tet[null] brains including β-lobe midline crosses and missing or misdirected α- and/or β-lobe(s) (Fig. 4*E*). Both phenotypes categorize as incompletely penetrant mushroom body defects with 30% of Tet[MI03920]/Tet[null] brains displaying midline fusion defects including different stages ranging from mild over moderate to complete fusion (Fig. 4*E*, left panel, open arrowheads), while 60% of Tet[MI03920]/Tet[null] brains displayed missing (white asterisks) or misdirected (empty arrowheads) α- and/or β-lobes (Fig. 4*E*, right panel). To conclude, our data show that the deficiency of dTet results in midline glia defects in developing brains. This midline glia phenotype is accompanied by axon commissure defects as well as misdirected and fused Fas2-positive axon tracts. Furthermore, axon guidance defects in Tet[MI03920]/Tet[null] adult brains manifest in two distinct incompletely penetrant mushroom body phenotypes including β-lobe midline crosses and missing or misdirected α- and/or β-lobe(s).

### dTet-deficient mutants display a highly significant increase in glial cell numbers in the brain lobes, accompanied by an increase in proliferating and apoptotic cells

**Figure 5.**
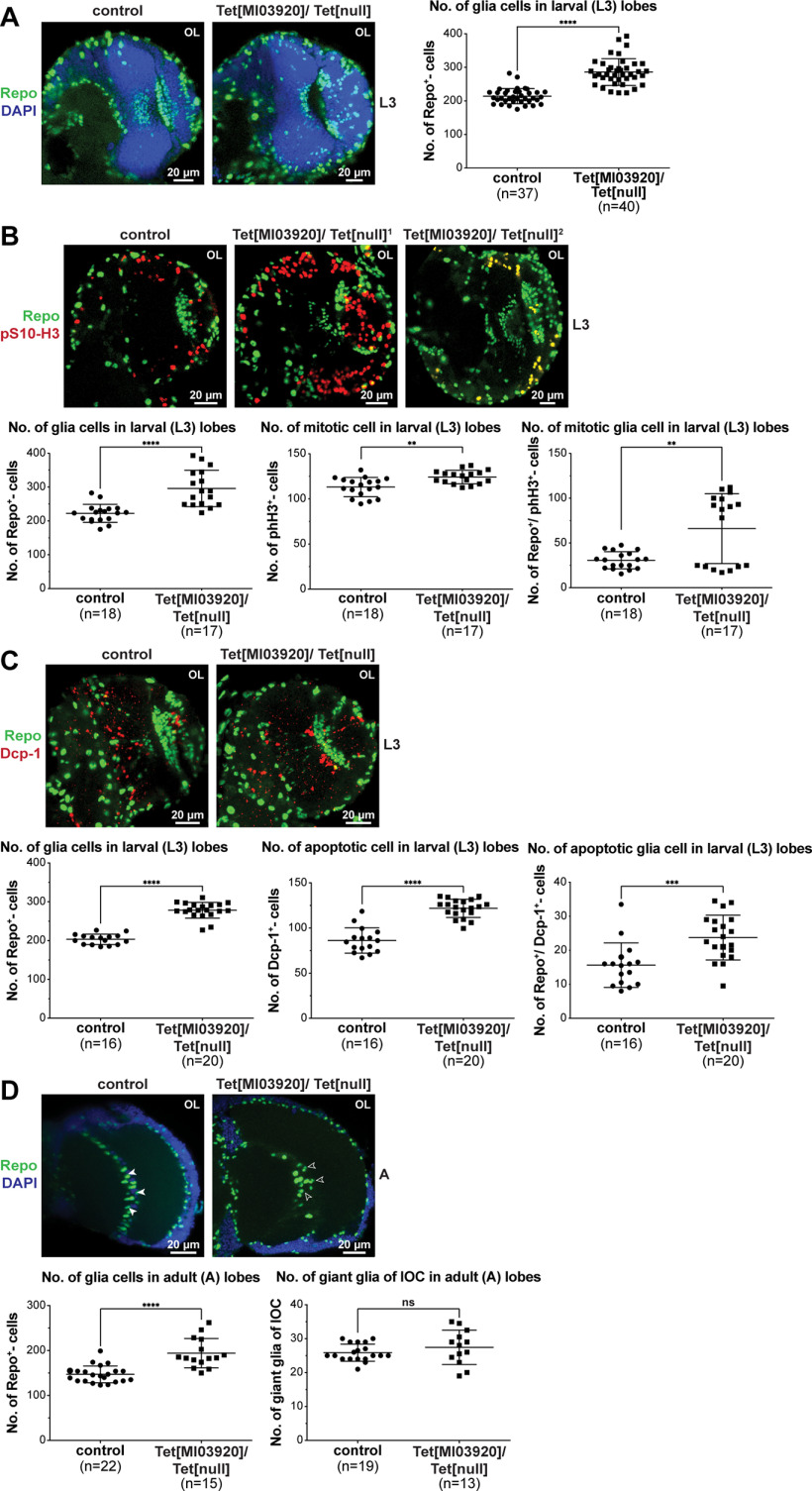
dTet-deficient mutants display a highly significant increase in glial cell numbers in the brain lobes, accompanied by an increase in proliferating and apoptotic cell numbers. Whole-mount control (wild-type w*^1118^*) or Tet[MI03920]/Tet[null] larval brains were dissected fixed and stained with (***A***) anti-Repo (glial cell marker), (***B***) anti-Repo and anti-phospho-Ser10 histone H3 (pS10-H3; mitotic marker), or (***C***) anti-Repo and anti-cleaved Death caspase-1 (Dcp-1; apoptosis marker). Whole-mount brains of 1-d-old control or Tet[MI03920]/Tet[null] adult flies were dissected fixed and stained with anti-Repo (***D***). Tet[MI03920]/Tet[null] adult brains exhibited scattered giant glial cells of the IOC (open arrowheads) as compared with linear alignment in control brains (white arrowheads). Displayed images are maximum intensity projections of 6 (1 μm) z-stacks taken with 40× oil objective. Cell counts were conducted using ImageJ plug-in ITCN. Each dot corresponds to the average amount of cells detected per animal in one brain lobe, where *n* indicates the number of animals analyzed. Statistical significance was analyzed by unpaired Student’s *t* test and graphs generated using GraphPad Prism version 5.01 (****p <* *0.0001; ns, not significant).

Next, we conducted a more detailed analysis of glia cell numbers and analyzed whether the balance between proliferation and apoptosis is affected in Tet[MI03920]/Tet[null] brain lobes. Intriguingly, we found that Tet[MI03920]/Tet[null] brains displayed a sharp and highly significant increase in glia cell numbers in the focal plane that contains the optic chiasm glia (Fig. 5*A*). The increase in glial cells did not appear to arise from a specific glia population in the brain lobes and was accompanied by a distinct increase of mitotic cells particular concentrated in the laminar region (Fig. 5*B*). Moreover, analysis of double positive cells (Repo^+^, phH3^+^) showed that in almost half of the investigated Tet[MI03920]/Tet[null] mutants, glial cells were the major proliferating cell type (Fig. 5*B*, Tet[MI03920]/Tet[null] 2), which was not observed in control brains (Fig. 5*B*, control) or the other half of Tet[MI03920]/Tet[null] brains (Fig. 5*B*, Tet[MI03920]/Tet[null] 1), where on average one quarter of the detected mitotic cells were glial cells. Next, we investigated the number of apoptotic cells in brain lobes of Tet[MI03920]/Tet[null] brains and found a highly significant increase in apoptotic cells (Dcp-1^+^ cells) compared with control brains. This overall gain in apoptotic cell numbers can partly be explained by the rise in apoptotic glial cells (Repo^+^, Dcp-1^+^), but to some extent appears to be because of an increase of apoptosis in nonglial cells (Fig. 5*C*). To investigate whether the increase in glial cell numbers observed in larval stages persists to the adult stage, we counted number of glial cells in optic lobes of 1-d-old adult Tet[MI03920]/Tet[null] brains. Indeed, we observed a highly significant increase of glial cells in adult Tet[MI03920]/Tet[null] brains compared with an age matched control group (Fig. 5*D*). Since dTet reporter is specifically expressed in the giant glia of the IOC, we also counted cell numbers of this specific glia population. While the counts of giant glial cells of IOC were comparable in Tet[MI03920]/Tet[null] brains (averaged 27) and controls (averaged 26; Fig. 5*D*, right graph) and in a similar range to previous reports (averaged 31, ranging from 25 to 37; Tix et al., 1997), we noticed that the giant glia of the IOC in Tet[MI03920]/Tet[null] brains were scattered and not arranged in a row in the middle of the optic lobe as observed for control brains (Fig. 5*D*, white vs open arrowheads). Notably, the giant glia of the outer and inner chiasm migrate during pupal stages outwards into their final position and the scattered appearance of the inner chiasm glia in Tet[MI03920]/Tet[null] brains indicates a possible migration defect.

### dTet-deficient mutants display a highly significant increase in glial cell numbers in the brain lobes that coincides with changes in hippo pathway activation

**Figure 6.**
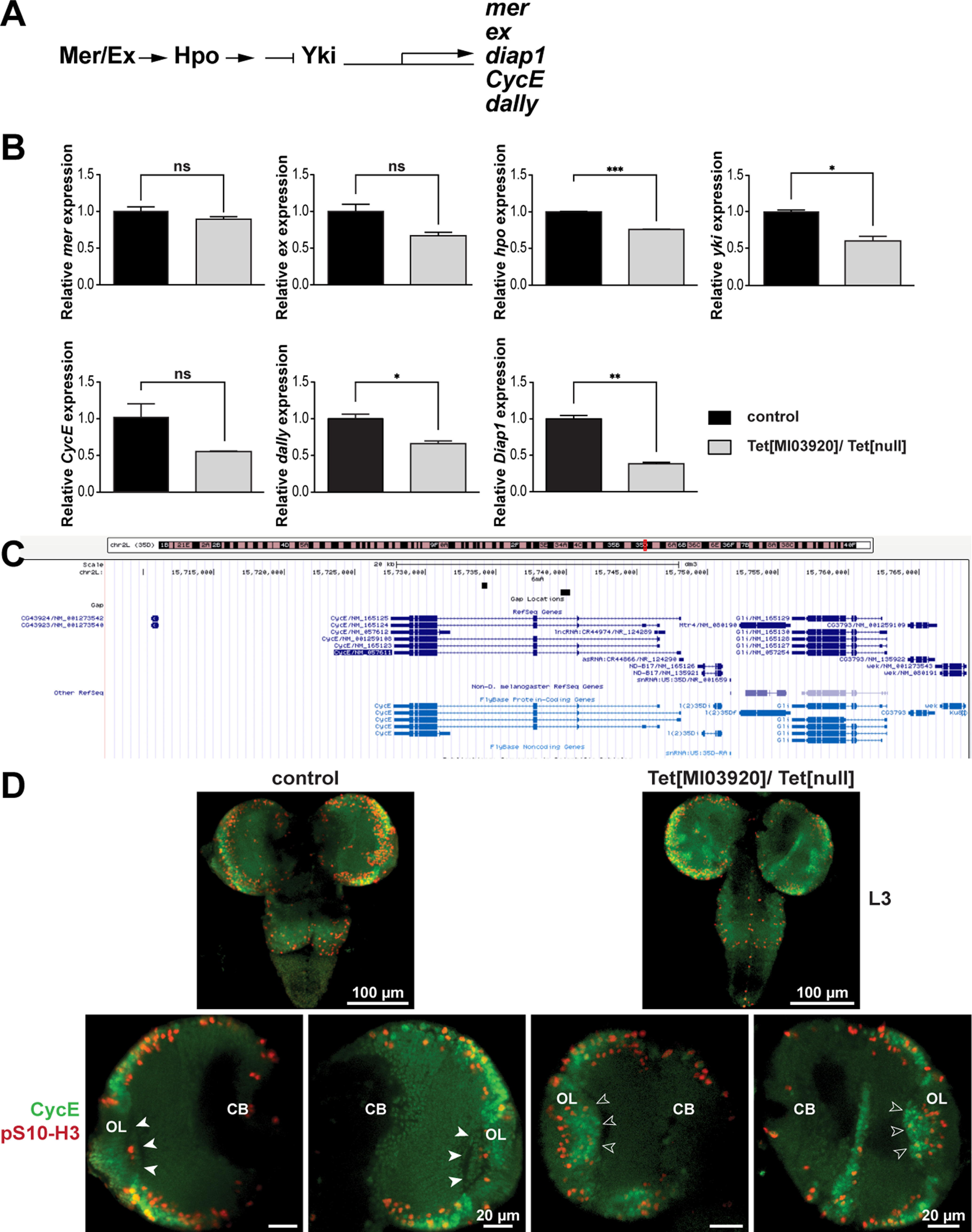
dTet-deficient mutants display a highly significant increase in glial cell numbers in the brain lobes that coincides with changes in hippo pathway activation. ***A***, Simplified schematic of the hippo signaling pathway, an intracellular kinase cascade that negatively regulates the transcriptional co-activator yki (yorkie), which in turn activates transcription of a wide range of downstream targets including mer (merlin), ex (expanded), diap1 (death-associated inhibitor of apoptosis 1), cycE (cyclin E), and dally (division abnormally delayed). Activation of the hippo pathway results in the downregulation of cell proliferation and upregulation of apoptosis. ***B***, Relative expression of selected hippo pathway members and several downstream targets listed in ***A***. Note that Tet[MI03920]/Tet[null] brains showed a 0.25-fold reduced transcription of hippo pathway member *hpo* that coincided with 0.4-fold reduced *yki* transcription. Additionally, downstream targets *dally* (0.35-fold) and *diap1* (0.69-fold) were both significantly reduced in Tet[MI03920]/Tet[null] brains. Hippo pathway members *mer* and *ex* that are acting upstream of *hpo* showed no significant change in expression in Tet[MI03920]/Tet[null] brains. ***C***, UCSC genome browser image showing two gain-of-6mA regions in the CycE genomic region in dTet-deficient brains relative to controls. These regions correspond to active 6mA demethylation loci in wild-type brains (Yao et al., 2018). ***D***, Whole-mount control (wild-type w*^1118^*) or Tet[MI03920]/Tet[null] larval brains were dissected fixed and stained with anti-CycE (Cyclin-E, control of cell cycle at G_1_/S transition) and anti-phospho-Ser10 histone H3 (pS10-H3). In control brains, cells in the lamina region do not show much CycE or pS10-H3 signal (white arrowheads), while the corresponding cells in Tet[MI03920]/Tet[null] brains show an increase in CycE and pS10-H3 signal (open arrowheads). Note that maximum z-projections are displayed for all markers. Statistical significance was analyzed by unpaired Student’s *t* test and graphs generated using GraphPad Prism version 5.01 (***p <* *0.001, **p <* *0.01, *p <* *0.05; ns, not significant).

To elucidate the molecular basis for the increase in glial cells in optic lobes, we decided to investigate changes in the Hippo signaling pathway known to control tissue size by downregulating cell proliferation and upregulating apoptosis (Fig. 6*A*; Reddy and Irvine, 2011; Staley and Irvine, 2012). Expression analysis by qPCR indicated that hippo (hpo) and yorkie (yki) expression is significantly reduced in Tet[MI03920]/Tet[null] brains compared with wild-type control brains, whereas merlin (mer) and expanded (ex) levels, that act upstream of hpo, were not affected. We then examined the expression of several downstream targets of the hippo pathway, whose transcription is activated by the transcriptional co-activator yki. While divion abnormally delayed (dally) and Death-associated inhibitor of apoptosis 1 (Diap1) were significantly downregulated in Tet[MI03920]/Tet[null] brains, Cyclin E (CycE) and ex were not significantly downregulated in Tet[MI03920]/Tet[null] brains (Fig. 6*B*). Since the role of hippo signaling in glial cell proliferation was mainly studied in optic lobes and eye discs (Reddy and Irvine, 2011), we wanted to investigate how CycE protein levels are altered in optic lobes of Tet[MI03920]/Tet[null] brains. CycE is a downstream target of the hippo pathway and was reported to be the most important cyclin in G_1_ to S phase transition, a critical point in the cell cycle, where the cell decides to either proliferate or differentiate (Richardson et al., 1993, 1995). Moreover, Yao and colleagues published that the genomic region of CycE exhibits two gain-of-6mA regions in dTet^null^ mutants relative to control flies (Fig. 6*C*) implicating that these regions may represent active 6mA demethylation loci in wild-type brains (Yao et al., 2018) that might be important for controlling *CycE* expression levels and thus cell proliferation in optic lobes. Intriguingly, CycE showed a slightly stronger signal in the lamina region of Tet[MI03920]/Tet[null] optic lobes that co-localized with the enhanced mitotic (pS10-H3) signal observed (Fig. 6*D*, white arrowheads vs empty arrowheads). The discrepancy of CycE quantities on mRNA and protein level might be because of the local increase of CycE protein levels in the laminar region of optic lobes that could not be detected in RNA extracted from whole brain.

In summary, Tet[MI03920]/Tet[null] brain lobes display a highly significant increase in glial cell numbers and the two mechanisms of cell survival and cell proliferation that usually balance cell numbers in the brain are both significantly altered in Tet[MI03920]/Tet[null] brains. Interestingly, in human brain tumors, reduction of hTETs has been observed to have an oncogenic effect (Orr et al., 2012). Additionally, lack of dTet resulted in disorganization of the giant glia of IOC reflected in their aberrant positioning rather than in altered cell quantity.

### Glia-specific knock-down of *dTet* has no significant effect on survival or locomotion, but knock-down of *dTet* in chiasm glia has a negative effect on survival

**Figure 7.**
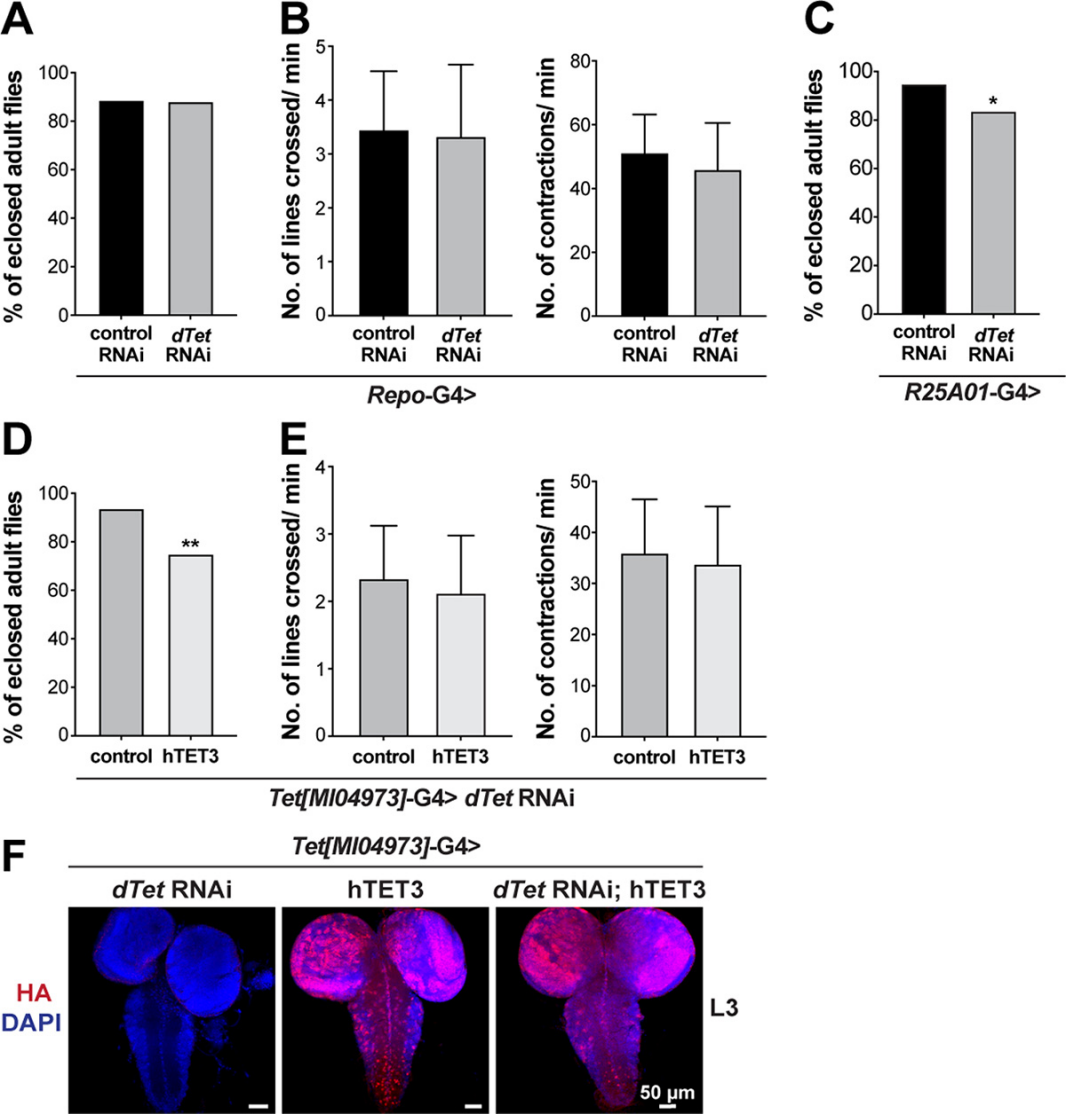
Glia-specific knock-down of *dTet* has no significant effect on survival or locomotion, but knock-down of dTet in chiasm glia has a negative effect on survival. ***A***, Knock-down of *dTet* in glial cells (*Repo*-Gal4) does not affect the survival from third instar larvae to adult stage (*n* = 80). ***B***, Crawling assays with wandering third instar larvae on 0.4-cm grid paper showed that knock-down of *dTet* in glial cells has no major effect on third instar larval locomotion or number of body contractions (*n* = 50). ***C***, Survival assay on third instar larvae yielded a moderate reduction in the number of eclosed adult flies, when *dTet* is specifically knocked down in the outer optic chiasm glial cells using driver *R25A01*-Gal4 [*p = 0.0209 (*n* = 80)]. ***D***, Survival assay on *Tet[MI04973]-*G4> *dTet* RNAi larvae that either co-expressed human TET3 or not, showed that simultaneous expression of hTET3 cannot rescue *dTet* knock-down. Larvae expressing *dTet* RNAi and hTET3 showed a significantly reduced survival rate [**p = 0.0011 (*n* = 80)]. ***E***, Crawling assay on *Tet[MI04973]-*G4> *dTet* RNAi larvae that either co-express human TET3 or not, showed no significant changes between both groups (*n* = 50). *GFP*-RNAi was used as a control. Statistical analysis on survival assays was performed by a chi-square. Statistical analysis on crawling assays was performed by unpaired Student’s *t* test. ***F***, Whole-mount *Tet[MI04973]-*G4> *dTet* RNAi, *Tet[MI04973]-*G4> hTET3 and *Tet[MI04973]-*G4> *dTet* RNAi; hTET3 larval brains were dissected fixed and stained with anti-HA antibody to validate expression of human TET3 transgene containing N-terminal Flag and HA tag (see Fig. 8*A*).

To further validate the role of dTet in glial cells we knocked down *dTet* using *Repo*-Gal4 driver. As a random RNAi control, we used GFP RNAi. We monitored survival of wandering third instar larvae to adults and also performed crawling assays to account for effects of *dTet* knock-down on locomotion and number of body contractions. Knock-down of dTet in glia cells did not show any significant effect on motility or contractions (Fig. 7*A*,*B*). Next, we knocked down *dTet* in the outer optic chiasm glial cells using driver *R25A01*-Gal4 and performed survival assay on third instar larvae. Knock-down of *dTet* in the outer optic chiasm glial cells resulted in a moderate reduction in the number of eclosed adult flies compared with control flies. Interestingly, midline glia-specific knock-down of *dTet* (*slit*-Gal4/*sim*-Gal4) has been published to be associated with survival and locomotion defects as well as defects in axon patterning (Ismail et al., 2019). Therefore, dTet might not be required for locomotion in all glia as it has been reported for neurons (Wang et al., 2018), but rather is only crucial in certain glia populations such as the midline glia and optic chiasm glia, where dTet is expressed. Finally, we performed survival and locomotion assay on *Tet[MI04973]-*G4> *dTet* RNAi larvae that either co-expressed hTET3 or did not express hTET3 (control). We observed that additional hTET3 expression had a negative effect on survival with only 75% of flies eclosing compared with 94% without hTET3 expression (Fig. 7*D*). Locomotion assays showed that hTET3 was not able to rescue the locomotion defects observed in *Tet[MI04973]-*G4> *dTet* RNAi larvae (Fig. 7*E*). The expression of hTET3 transgene containing N-terminal HA tag through *Tet[MI04973]-*G4 driver alone and in combination with *dTet* RNAi was validated by staining of larval brains with HA antibody (Fig. 7*F*).

### Expression of human TET3 in dTet-expressing cells results in an increase in glial cells in larval brain lobes as well as reduced life span and a circadian phenotype in adult flies

**Figure 8.**
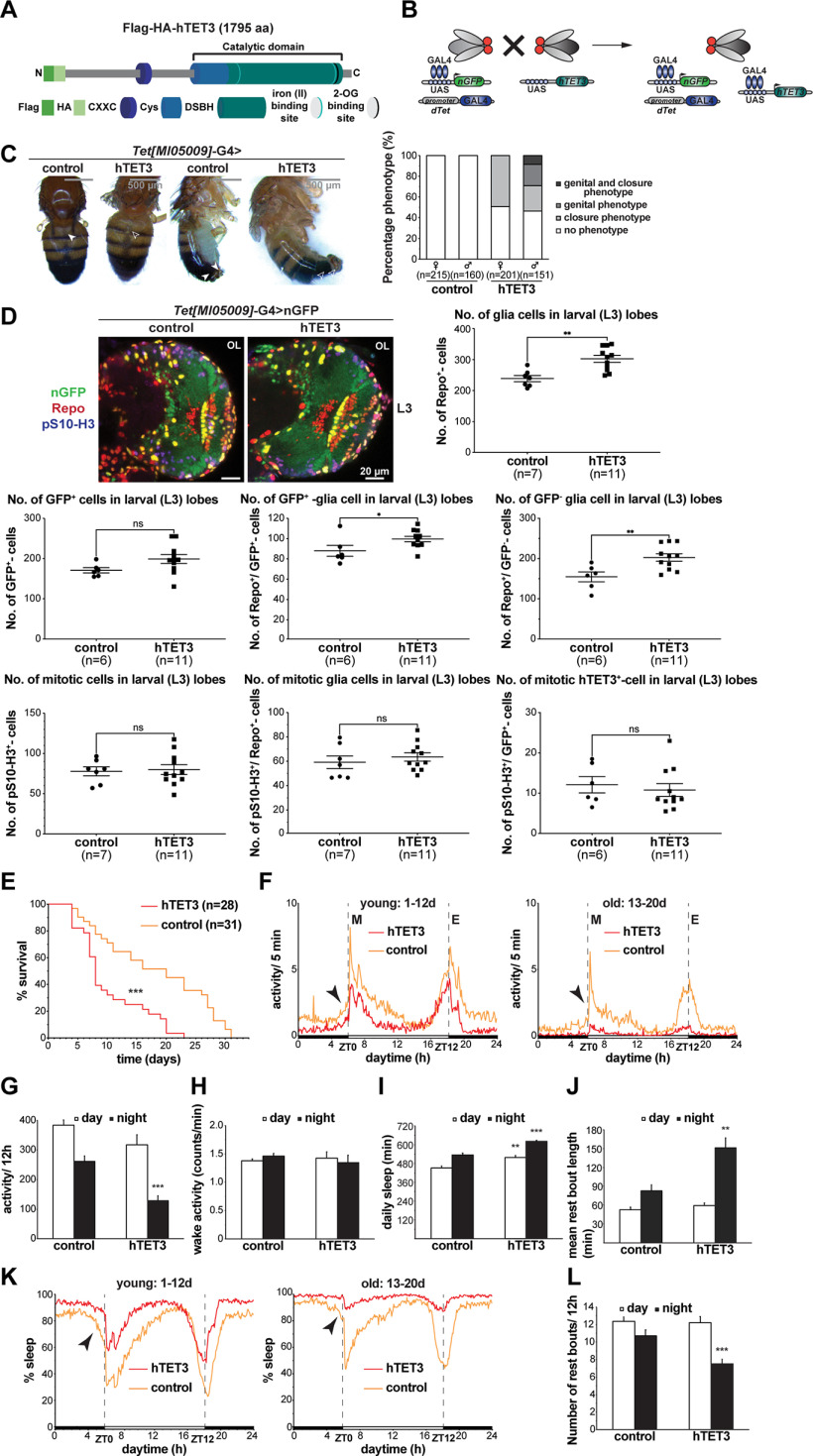
Expression of human TET3 in dTet-expressing cells results in an increase in glial cells in larval brain lobes as well as a reduced life-span and a circadian phenotype in adult flies. ***A***, Schematic representation of the human TET3 transgene with N-terminal Flag and HA tag used in the described experiments. Functional domains are indicated including a CXXC zinc finger, a Cys-rich domain and a double-stranded β helix (DSBH) domain containing iron (II) and 2-OG binding sites. ***B***, The Gal4/UAS system was used for targeted expression of human TET3 (hTET3). The system is composed of two independent parent transgenic lines, the *Tet[MI05009]*-G4 driver line, in which the *Gal4* gene is expressed in a *dTet*-specific pattern, and the hTET3 transgene containing line that contains the Gal4 DNA binding sequence UAS (upstream activating sequence) adjacent to the *hTET3* gene. Mating of the described parental flies results in a F1 generation, where Gal4 is expressed in the transcriptional pattern of *dTet* and binds to the UAS to activate transcription of hTET3 in the same pattern. Here, the Gal4 driver was combined with a UAS-containing transgene to express nuclear GFP (nGFP) to visualize the cells/tissue that express hTET3 transgene. ***C***, Light microscope images of control flies and *Tet[MI05009]*-G4> hTET3-expressing flies displaying incomplete fusion of the adult abdominal epidermis (open arrowhead, right panel) as well as protrusions of the most posterior abdominal segments including the male genitalia (open arrowhead, left panel). Approximately 50% of flies displayed either of the phenotypes or both as shown in the sex-specific quantification in the left graph. ***D***, Whole-mount *Tet[MI05009]*-G4::UAS-nGFP> control or *Tet[MI05009]*-G4::UAS-nGFP> hTET3 larval brains were dissected fixed and double stained with anti-Repo and anti-pS10-H3. Displayed images are maximum z-projections of six (1 μm) z-stacks taken with 40× oil objective. Cell counts were conducted using ImageJ plug-in ITCN. Each dot corresponds to the average amount of cells detected per animal in one brain lobe, where n indicates the number of animals analyzed. Statistical significance was analyzed by unpaired Student’s *t* test and graphs generated using GraphPad Prism version 5.01 (**p <* *0.005, *p <* *0.05, ns, no statistical significance). ***E***, Kaplan–Meier survival curve of male flies expressing either no transgene (driver control, *n* = 31) or hTET3 (*n* = 28) through *Tet[MI05009]*-G4. Statistical significance of difference between survival curves was determined using the Mantel–Haenszel test (*p *<* *0.005). ***F***, Activity graphs illustrating daily locomotor activities of flies over several days. For each group, the locomotor activity levels of individual flies (*n* ≅ 30) were measured in 5-min bins and then averaged to obtain a representative activity profile. Since locomotion is age-dependent, we subdivided flies in two age groups: “young”: 1–12 d old and “old”: 13–20 d old. *Drosophila melanogaster* generally exhibits two activity bouts one centered around ZT0 (morning peak) and the second around ZT12 (evening peak). Black arrows indicate the anticipatory increase in locomotor activity that occurs before light transition states. ***G***, Graph showing average locomotor activity over 12-h intervals. Note that hTET3-expressing flies are significantly less active at night. Statistical significance was analyzed by unpaired Student’s *t* test (***p <* *0.0005). ***H***, Graph illustrating the wake activity in counts per min over 12-h intervals. Wake activity, is a measure of the activity rate when the flies are awake. Note that the wake activity is comparable between hTET3-expressing and control flies indicating that hTET3 flies are affected in sleep/rest behavior and not in locomotion. ***I***, Graph showing the average of daily sleep minutes for all flies in one group for 12-h intervals (day: light on, night: light off) over 20 d. During day and night hTET3-expressing flies showed a significant increase in sleep time compared with control flies. Statistical significance was analyzed by unpaired Student’s *t* test (**p <* *0.005, ***p <* *0.0005). ***J***, Graph indicating the mean rest bout length of each group in minutes for 12-h intervals. The mean rest bout length is a measure of how consolidated sleep is and was significantly higher for TET3-expressing flies during night time. Statistical significance was analyzed by unpaired Student’s *t* test (**p <* *0.005). ***K***, Graph illustrating the percent of time that flies spend sleeping over several days. For each group, the percent of flies sleeping was measured in 5-min bins and then averaged to obtain a representative sleep profile. Since sleep is age-dependent, we subdivided flies in two age groups as described above. ZT0 indicates morning peak and ZT12 the evening peak. Black arrows indicate the anticipatory phase occurring before light transition states. ***L***, Graph showing the average number of rest bouts for all flies in one group for 12-h intervals over 20 d. During night hTET3-expressing flies showed significantly less rest bouts compared with control flies. Statistical significance was analyzed by unpaired Student’s *t* test (***p <* *0.0005). ZT stands for Zeitgeber time. ZT0 indicates the beginning of the day (light phase) and ZT12 the beginning of the night (dark phase).

After establishing the effects of reducing *Drosophila* Tet on phenotype, survival, and glial cell proliferation status, we wanted to investigate the effects of overexpressing human TET in the fly (Fig. 8*A*,*B*). For this, we used human TET3, since its catalytic domain was proposed to be the closest to dTet’s catalytic domain in terms of structural similarity (Dunwell et al., 2013). Moreover, TET3 is the only mammalian TET homolog essential for embryonic development (Gu et al., 2011) and *Tet3* transcript is the most abundant *Tet* transcript in the brain (Szwagierczak et al., 2010). First, we checked adult flies expressing TET3 through the *Tet[MI05009]*-G4 promoter for any obvious phenotypic alterations. We noticed two specific phenotypes that appeared in about half of TET3-expressing flies and were not observed in the driver control group (Fig. 8*C*). The first observed phenotype was incomplete fusion of the adult abdominal epidermis (49% of females and 33% of males), and the second was protrusion of the most posterior abdominal segments including the male genitalia that was male specific (29%, with 9% of males displaying both phenotypes; Fig. 8*C*). Next, we investigated the effects of expressing TET3 through *Tet[MI05009]*-G4 line on the glia population in larval brain lobes. For this, we analyzed (1) number of glial cells, (2) number of proliferating cells, (3) number of TET3-expressing cells, and (4) overlap between these three cell populations in brain lobes of third instar larvae. Surprisingly, we detected a highly significant increase in number of glial cells on TET3 expression accompanied by a slight increase in GFP-positive cells (Fig. 8*D*). Analysis of GFP positive and negative glial cell populations, which correspond to the glia population expressing TET3 and the glia population not expressing TET3, respectively, showed, that both glial cell populations (Repo^+^, GFP^+^) and (Repo^+^, GFP^-^) are significantly increased in TET3-expressing larvae (Fig. 8*D*, lower left panel) indicating that TET3 has not only an autonomous, but also a nonautonomous effect on glial cell numbers. Notably, the increase in GFP^+^-glial cells was mainly observed in the medulla area, whereas the increase in GFP^-^-glial cells appeared to be mainly originating from the lamina region. Next, we checked whether the increase in glial cells coincides with an increase in proliferation by staining for the mitosis marker pS10-H3. However, we neither detected an overall increase in mitotic cells, nor an increase in mitotic glial cells, nor an increase in mitotic TET3-expressing cells (phH3^+^, GFP^+^; Fig. 8*D*, lower panel), suggesting that the observed increase may be because of factors other than proliferation such as changes in cell survival or differentiation.

We next set out to determine effects of TET3 expression on adult life-span, changes in behavioral parameters including activity and sleep characteristics as well as changes in circadian rhythm. For this, a DAM system was used under a standard 12 h lights on/12 h lights off constant 25°C temperature regime. Flies expressing TET3 through *Tet[MI05009]*-G4 displayed significantly reduced life-span of maximal 23 d (median survival 8 d) compared with the control group, *Tet[MI05009]*-G4 flies crossed to wild-type flies, that lived for maximal 31 d (median survival 20 d; Fig. 8*E*). In addition, we analyzed circadian parameters by looking at average locomotor activities of two age groups, where 1- to 12-d-old flies are considered “young” and 13- to 20-d-old flies are considered “old.” Both, TET3-expressing and control flies, exhibited two major activity peaks, the first centered around Zeitgeber time 0 (ZT0), the beginning of the light phase, the so-called morning peak, and the second around Zeitgeber time 12 (ZT12), the beginning of the dark phase that is called the evening peak (Fig. 8*F*). Compared with control flies, TET3-expressing flies showed a lack of anticipatory increase in locomotor activity before the dark-to-light and light-to-dark transition indicating that TET3 expression may interfere with proper endogenous clock function. Interestingly, dTet has been previously linked to controlling the development of pigment-dispersing factor (PDF)-expressing neurons which are essential for the circadian rhythm, in particular the morning activity (Wang et al., 2018). While the total activity of TET3-expressing flies was significantly less during the night compared with control flies (Fig. 8*G*), the wake activity, which is a measure of the activity when flies are awake, was comparable between TET3-expressing and control flies (Fig. 8*H*). This indicates that TET3-expressing flies are actually affected in sleep/rest behavior and do not just display impaired locomotion. We found that the amount of sleep during light phase (day) and dark phase (night) was significantly higher in TET3-expressing flies (Fig. 8*I*,*K*), particularly in older flies (Fig. 8*K*). The mean rest bout length which is a measure of how consolidated the sleep is, was significantly longer for TET3-expressing flies at night, but not affected during the day (Fig. 8*J*). Accordingly, the number of rest bouts for TET3-expressing flies was significantly less at night (Fig. 8*L*), indicating that these flies display longer sleep phases.

### Expression of human TET3 in *Drosophila* glial cells results in circadian phenotypes and decrease in mitotic cells

**Figure 9.**
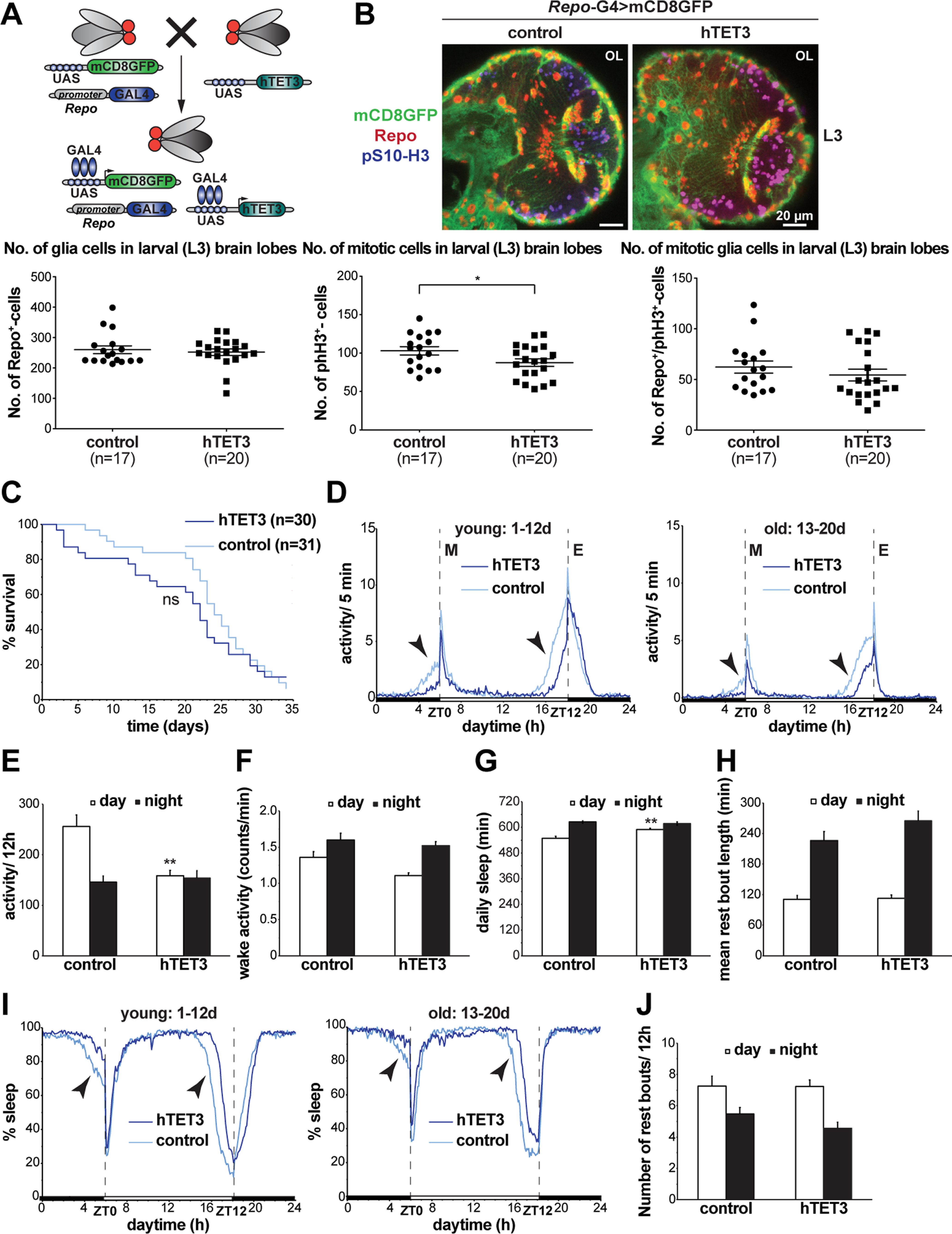
Expression of human TET3 in glial cells results in circadian phenotypes and decrease in mitotic cells. ***A***, The Gal4/UAS system was used for targeted expression of human TET3. The system is composed of two independent parent transgenic lines, the *Repo*-Gal4 driver line, in which the *Gal4* gene is expressed in a glia-specific pattern, and the hTET3 transgene containing line that contains the Gal4 DNA binding sequence UAS (upstream activating sequence) adjacent to the *hTET3* gene. Mating of the described parental flies results in a F1 generation, where Gal4 is expressed in the transcriptional pattern of the glia marker *Repo* and binds to the UAS to activate transcription of hTET3 in the same pattern. Here, the Gal4 driver was combined with a UAS-containing transgene to express membrane-targeted GFP (mCD8-GFP) to visualize the cells/tissue that express hTET3 transgene. ***B***, Whole-mount *Repo*-Gal4::UAS-mCD8GFP> control or *Repo*-Gal4::UAS-mCD8-GFP> hTET3 larval brains were dissected fixed and double stained with anti-Repo and anti-pS10-H3. Displayed images are maximum z-projections of six (1 μm) z-stacks taken with 40× oil objective. Cell counts were conducted using ImageJ plug-in ITCN. Each dot corresponds to the average amount of cells detected per animal in one brain lobe, where n indicates the number of animals analyzed. Statistical significance was analyzed by unpaired Student’s *t* test and graphs were generated using GraphPad Prism version 5.01 (*p <* *0.05; ns, *p *>* *0.05). ***C***, Kaplan–Meier survival curve of male flies expressing either no transgene (driver control, *n* = 31) or hTET3 (*n* = 30) through *Repo*-Gal4. Statistical significance of difference between survival curves was determined using the Mantel–Haenszel test (*p *<* *0.005). ***D***, Activity graphs illustrating daily locomotor activities of flies over several days. For each group, the locomotor activity levels of individual flies (*n* ≅ 30) were measured in 5-min bins and then averaged to obtain a representative activity profile for two age groups as described above. *Drosophila melanogaster* generally exhibits two activity bouts one centered around ZT0 (morning peak) and the second around ZT12 (evening peak). Black arrows indicate the anticipatory increase in locomotor activity that occurs before light transition states. ***E***, Graph showing average locomotor activity over 12-h intervals. Note that hTET3-expressing flies are significantly less active at night. Statistical significance was analyzed by unpaired Student’s *t* test (***p <* *0.0005). ***F***, Graph illustrating the wake activity counts per minute over 12-h intervals. ***G***, Graph showing the average of daily sleep minutes for all flies in one group for 12-h intervals (day: light on, night: light off) over 20 d. During day and night hTET3-expressing flies showed a significant increase in sleep time compared with control flies. Statistical significance was analyzed by unpaired Student’s *t* test (**p <* *0.005, ***p <* *0.0005). ***H***, Graph indicating the mean rest bout length of each group in minutes for 12-h intervals. The mean rest bout length is a measure of how consolidated sleep is and was significantly higher for TET3-expressing flies during night time. Statistical significance was analyzed by unpaired Student’s *t* test (**p <* *0.005). ***I***, Graph illustrating the percent of time that flies spend sleeping over several days. For each group, the percent of flies sleeping was measured in 5-min bins and then averaged to obtain a representative sleep profile. ZT0 indicates morning peak and ZT12 the evening peak. Black arrows indicate the anticipatory phase occurring before light transition states. ***J***, Graph showing the average number of rest bouts for all flies in one group for 12-h intervals over 20 d. Note that the number of rest bouts are comparable between hTET3-expressing and control flies. ZT stands for Zeitgeber time. ZT0 indicates the beginning of the day (light phase) and ZT12 the beginning of the night (dark phase).

Since *Tet[MI05009]*-G4 drives expression in neurons and glia populations (Fig. 2*A*,*B*), we continued by expressing TET3 using the glia-specific driver *Repo*-Gal4 to understand which aspects of the observed phenotypes are glial specific. In addition, the *Repo*-Gal4 driver used was combined with UAS-mCD8-GFP, a membrane-targeted green fluorescent protein, to investigate whether TET3 would induce changes in cell migration or morphology (Fig. 9*A*). We also analyzed whether glial cell numbers, mitotic cell numbers or number of mitotic glial cells changed in amount, morphology, or position on *Repo*-Gal4-driven TET3 expression. The number of glial cells was unaltered in TET3-expressing larval brain lobes and no significant change was detected in mitotic glial cell numbers; however, there was a significant decrease in mitotic cells (Fig. 9*B*).

Adult flies expressing TET3 under the *Repo*-Gal4 promoter displayed no obvious phenotypic alterations (data not shown). As described above, a DAM system was used under the same conditions to determine effects on adult life-span and changes in behavioral parameters including activity and sleep characteristics as well as changes in circadian rhythm. Flies expressing TET3 under the *Repo*-Gal4 promoter appeared to die to an increased degree in the first 20 d, but the difference was not significant when compared with the control group. Moreover, both groups had few surviving flies after 35 d. Therefore, TET3 expression in all glial cells did not affect overall survival (Fig. 9*C*). Analysis of average locomotor activities of young (1–12 d) and old flies (13–20 d) showed that both, TET3-expressing and control flies, exhibited two major activity peaks around ZT0 and around ZT12 (Fig. 9*D*). As observed with *Tet[MI05009]*-G4-driven expression, glia-specific TET3 expression resulted in a lack of anticipatory increase in locomotor activity before the dark-to-light and light-to-dark transition indicating that TET3 expression in the glia population is sufficient to induce this circadian phenotype (Fig. 9*D*). While the total activity of TET3-expressing flies was significantly less during the day compared with control flies (Fig. 9*E*), the wake activity was not significantly reduced in TET3-expressing flies (Fig. 9*F*). Similar to *Tet[MI05009]*-G4-driven expression, the amount of sleep during light phase (day) was significantly higher in TET3-expressing flies (Fig. 9*G*,*I*), this effect was more obvious in older flies (Fig. 9*I*). However, neither the mean rest bout length (Fig. 9*H*) nor the amount of rest bouts (Fig. 9*J*) was affected in those flies, suggesting that both, *Tet[MI05009]*-G4 and glia-specific TET3 expression, affects the sleep/rest behavior, but to different degrees. To summarize, while glia-specific expression of TET3 had no effects on morphology and survival, we observed effects on the sleep/rest cycle and the anticipatory activity, indicating that this circadian phenotype might be caused by TET3 expression from the glia population.

## Discussion

TET proteins are important regulators of cellular identity and loss of function is associated with cancer onset and progression. Early studies identified *TET1* as a partner gene undergoing fusion with the mixed lineage leukemia (*MLL*) gene in rare cases of acute myeloid leukemia (AML) and acute lymphocytic leukemia (ALL; Ono et al., 2002; Lorsbach et al., 2003). Since then, numerous studies demonstrated that TET proteins are frequently mutated in cancer (Huang and Rao, 2014; Han et al., 2015; Scourzic et al., 2015). Although mutations in the *TET* gene family rarely occur in solid tumors (COSMIC database: 1–3%), downregulation of *TET* expression has been observed in diverse solid tumors including lung, breast, colon, skin and brain cancers (Huang and Rao, 2014). Notably, TET-mediated DNA demethylation has been reported to play an important role in brain tumors, particularly in gliomas (Bian et al., 2014). For example, a frequent mutation in gliomas, isocitrate dehydrogenase (IDH) 1 and 2 results in inhibition of all 2OG-dependent dioxygenases, including TET protein’s ability to catalyze 5mC to 5hmC (Yen et al., 2010; Xu et al., 2011; Huang and Rao, 2014). In recent years, several studies embarked on understanding the role of TET in brain development and behavior, especially with the emergence of functional studies in *Drosophila melanogaster* targeted toward understanding how this single demethylase (dTet) affects brain development and regulates gene transcription (Zhang et al., 2015; Delatte et al., 2016; Yao et al., 2018; Ismail et al., 2019). As reported previously, the dTet-deficient alleles used in this study showed survival disadvantages with only 40–60% of flies eclosing. This partial lethality is accompanied by aberrant larval locomotion, escalating to adult immobility, and eventually death within 2 d posteclosion. The lethality observed at pupal stage might be attributed to early requirement of dTet in embryonic and larval cells and tissues. dTet expression was previously reported in both, embryonic Mef-2-positive mesodermal cells and embryonic neuronal cells, albeit expression was lower in mesodermal muscle precursor cells (Wang et al., 2018; Ismail et al., 2019). However, lethality could also be because of requirement of dTet in other discs and imaginal tissues such as wing and eye discs (Wang et al., 2018). dTet expression has been previously reported in developing larval brains, in both, postmitotic neurons and differentiated glial cells (Delatte et al., 2016; Wang et al., 2018; Ismail et al., 2019). Here, we show that glial expression of dTet is not restricted to the central brain region (larval/adult) and midline glia (larvae), but we identified a unique population of glial cells in the optic lobe known as chiasm glia, in addition to scattered expression in optic lobes. Chiasm glia are differentiated early during larval development and are required for optic lobe development by providing guidance to outgrowing axons in the lamina region of the optic lobe (Shinomiya et al., 2019). Proper glia organization in the optic lobe and chiasm is essential for adequate motion detection circuits in flies (Jeffery, 2001). A previous study has reported that slit expression by midline glia may be involved in axonal midline crossing defects (Ismail et al., 2019). Interestingly, slit is also expressed by the optic chiasm glia (Tix et al., 1997; Caipo et al., 2020). However, whether a similar mechanism is involved in optic neuronal axon guidance and whether this affects fly behavior and motion detection capabilities needs to be further investigated. Here, we have characterized an additional axonal defect, which manifests in the longitudinal axons in the lower VNC. The VNC appeared narrower and clustered as if longitudinal axons are fused together in dTet mutants. This goes in parallel with axon divergence seen in the VNC across the midline glia emphasizing a more general role of dTet in proper axonal guidance in the VNC.

Expression of mammalian TETs has been mainly reported in neuronal cells (Antunes et al., 2019). While one study reported that TET1 is expressed in astrocytes in adult mouse hippocampus at low levels (Kaas et al., 2013), another study analyzed TET protein expression during oligodendrocyte development *in vivo* and *in vitro* and found a dynamic pattern of TET protein expression that is accompanied by dynamic changes in 5hmC levels during oligodendrocyte maturation (Zhao et al., 2014). In addition, knock-down experiments from the same study demonstrated that all three TET enzymes are required for normal oligodendrocyte development (Zhao et al., 2014). To date, data available on TET protein expression and function in glial cells remains scarce and this study on dTet requirement in glia of the adult/larval optic chiasm and midline of developing larvae may provide some clues for similar functions of TETs in glia in higher organisms as both glia types are anatomically conserved in vertebrates. Our findings indicate the importance of dTet in glia hemostasis as glial cell numbers were significantly increased in brain lobes of Tet[MI03920]/Tet[null] larvae coinciding with an increase in the mitotic index. We further investigated CyE patterns in brain lobes. CycE is a downstream target of the hippo pathway and is the most important cyclin in G_1_ to S phase transition in *Drosophila* (Richardson et al., 1993, 1995). This is of importance as it may explain the observed expansion in glial cell population in Tet[MI03920]/Tet[null] flies. The hippo pathway has been shown to maintain the quiescence in *Drosophila* neural stem cells and any perturbation in this pathway may affect glia as well as neuronal differentiation and proliferation (Ding et al., 2016). Although neoplastic brain is one of the standard brain phenotypes assessed in *Drosophila* glioblastoma models (Read, 2011), deficiency of dTet did not cause neoplasia as is the case when expressing common glioblastoma EGFR-Ras and PI3K mutations in *Drosophila* glia (Read, 2011); however, it caused an expansion in a differentiated glia population (Repo positive cells) in larval brain lobes. It remains interesting to identify whether this increase in glia numbers is accompanied by loss of neurons, since we detected an increase in apoptotic cells that was not of glial origin. There are different glial cells in the developing *Drosophila* brain, such as cortex glia, surface glia, neuropil glia and peripheral glia. Each responsible for specific functions as is the case in vertebrates’ astrocytes, oligodendrocytes, microglia, and Schwann cells, respectively (Freeman and Doherty, 2006). Assessing which glia population is proliferating on loss of dTet, will determine which glia processes dTet safeguards in the *Drosophila* brain. Here, we show for the first time that dTet is playing a more general role in axon guidance in the VNC and that dTet is required for proper glial cell differentiation in the brain lobes. Additionally, a recent study described defects in mushroom bodies, a region in the fly brain that is well characterized and associated with olfactory learning and memory (Heisenberg, 2003), on neuron-specific knock-down of dTet (Yao et al., 2018). We have confirmed and analyzed these phenotypes in more detail with our dTet-deficient allele further reinforcing a role for dTet in olfactory learning and memory. In adult mammalian brains, TET proteins have emerged as important players in modulating neuronal plasticity, behavior and memory (Antunes et al., 2019); however, their exact roles in brain function appear to be somehow distinct and region-dependent. TET1, for example, has been reported to be regulated by neuronal activity in mice, where it positively regulates several genes implicated in learning and memory. Moreover, its overexpression impaired hippocampus-dependent long-term associative memory independent from its catalytic activity (Kaas et al., 2013). TET2 on the other hand has been linked to neurogenic rejuvenation. Conditional knock-out of TET2 within the hippocampal neurogenic niche of young mice led to decreased neurogenesis and impairment of learning and memory, whereas overexpression of TET2 in the same neurogenic niche of mature adults reversed age-related decline in neurogenesis and enhanced learning and memory (Gontier et al., 2018). Based on the described glia phenotypes in Tet[MI03920]/Tet[null] flies, we wanted to investigate whether expressing TET3, the closest human homologues of dTet, in *Drosophila*, would recapitulate some glioma like phenotypes. hTET3 expression in combination with *dTet* knock-down, did show a negative effect on survival and hTET3 was not able to rescue the *dTet* knock-down associated locomotion defects. It is therefore possible, that the amount of TET3 expressed, and cell-specific expression, might be key factors in restoring some of the functional defects observed in dTet-deficient flies. Expressing human TET3 in dTet-expressing cells caused a general increase in glial cells in the optic lobes. This increase was seen in both, dTet positive and negative cells, indicating that TET3 has not only an autonomous, but also a nonautonomous effect on glial cell proliferation. Interestingly, this increase in glia proliferation was not observed when TET3 was expressed only in differentiated glia populations (Repo driver) indicating that TET3 might have an earlier effect at the stem cell level, which is usually observed in cancer. Although all three TET enzymes are expressed in the central nervous system of mice and human, TET3 has been shown to be the most abundant transcript in different mammalian brain regions (Szwagierczak et al., 2010). A study on mouse embryonic stem cells (mESCs), either lacking *Tet3* alone or with triple deficiency of *Tet1/2/3*, found that TET proteins, and in particular TET3, play a key role in modulating Wnt signaling and establishing the proper balance between neural and mesodermal cell fate specification in ESCs as well as in mouse embryos (Li et al., 2016). Furthermore, a recent study in hepatoblastoma (embryonal liver tumor) re-emphasized that not only loss of TET function, but also aberrant expression of TET can lead to DNA hypomethylation and an increase in overall 5hmC level in these tumors (Rivas et al., 2019). Another study reported that TET3 expression is activity-dependent in primary cortical neurons and mediates accumulation of 5-hmC, in turn promoting gene expression and rapid behavioral adaptation contributing to formation of fear extinction memory, an important form of reversal learning (Li et al., 2014). In our *Drosophila* human TET3 model, expression of TET3 caused premature mortality in adult flies accompanied by an increase in glial cells in larval brain lobes, only when expressed in dTet expressing tissues. In contrast, defects in circadian behavior were observed on expression of TET3 in both, dTet expressing tissues as well as differentiated glial cells. Notably, dTet has previously been linked to the circadian rhythm. In particular, dTet has been shown to be required during embryonic and larval stages in PDF neurons to ensure proper circadian behavior in adult flies (Wang et al., 2018). In fact, the circadian rhythm also modulates the timing of preadult developmental events in *Drosophila* (Yadav et al., 2014; Varma et al., 2019) and thus, defects in the circadian clock might contribute to the observed lethality.

Overall, our study reports a yet undescribed role for dTet in normal glia homeostasis, proper arrangement in optic chiasm as well as behavior. Finally, we establish a human TET3 *Drosophila* model, and show that ectopic expression of TET3 results in deregulation of glia proliferation in the optic lobe and affects fly survival and circadian rhythm.

**Table 1:** List of Primers used in real-time PCR

Gene	Primer sequence	Temperature (°C)
*RpL11*	Forward: 5′-CGATCCCTCCATCGGTATCT-3′ Reverse: 5′-AACCACTTCATGGCATCCTC-3′	57°C
*Fife*	Forward: 5′-GGAGGACATCCAGAACGACG-3′ Reverse: 5′-GCTATATCGGGTAGCTGCGA-3′	57°C
*Mrtf*	Forward: 5′-GAGTCAGCACGTCACTGGAA-3′ Reverse: 5′-GTCGCGTATTCGACGTGTTG-3′	57°C
*Ahcyl*	Forward: 5′-CTAAAGGGGCAGGGCTGCAT-3′ Reverse: 5′-TGTCTACGTTGCGGATCACC-3′	57°C
*CG12093*	Forward: 5′-CACAGGATACTTGGCAGGGT-3′ Reverse: 5′GCAAGATTGCTCTTGCCATCA-3′	57°C
*Spz5*	Forward: 5′-TACGGAAGCACCCACGTATC-3′ Reverse: 5′-GTGACCGCCAGAAGATGAGG-3′	57°C
*dTet*	Forward: 5′-AATGCTGGACAAATTTCCCT-3′ Reverse: 5′-CCTTCGATTTGCTACTAGCTC-3′	57°C
*yorkie*	Forward: 5′-AAGTGGACGGGGATAGCCA-3′ Reverse: 5′-CGCTGCGCGAAAGGAACTG-3′	58°C
*merlin*	Forward: 5′-CGGTGCAAACAATCGACCATC-3′ Reverse: 5′-GTCCTTCTGGTGCTCCTGTG-3′	58°C
*hippo*	Forward: 5′-CTGGAGTCGAACTTGGGCAC-3′ Reverse: 5′-GGCCATCTCCCGCATTTTTG-3′	58°C
*cyclin E*	Forward: 5′-TAGCCGTGAAAAAGACCGCA-3′ Reverse: 5′-CTGCTGGTAGAACAAACACTCTTG-3′	58°C
*Diap-1*	Forward: 5′-ATCGTGGTGCGATAAGAGGTG-3′ Reverse: 5′-TCCTCTGAAGTCGAAACAGAGACA-3′	58°C
*expanded*	Forward: 5′-AGAGTGCACGAAGAGTGAGC-3′ Reverse: 5′-CGCGTTCGAAATACGTTGCTTA-3′	58°C
*dally*	Forward: 5′-ACCATCGTGGACAACTTCTGC-3′ Reverse: 5′-GGTCTGAGCCTTCGCATTGA-3′	58°C

## Synthesis

Reviewing Editor: Silvia Pagliardini, University of Alberta Decisions are customarily a result of the Reviewing Editor and the peer reviewers coming together and discussing their recommendations until a consensus is reached. When revisions are invited, a fact-based synthesis statement explaining their decision and outlining what is needed to prepare a revision will be listed below. The following reviewer(s) agreed to reveal their identity: NONE.

The authors in the current manuscript took the comments and suggestions from the reviewers and used another dTet-Gal4 line. They confirmed the dTet expression in the optic lobe and found human Tet expression has a negative effect on dTet knockdown experiment. Overall, the quality of the manuscript improved although the authors should carefully proofread the manuscript since there are several typos and mistakes.

1, The authors found that additional hTET3 expression can not rescue the survival of dTet knock down flies. It suggests that the function of human Tet3 and dTet might not be conserved. Have the authors checked the expression level of hTET3?

2, The behaviors in Figure 8 are not very convincing to me, especially in those old experimental lines. They are about to die around day 20.

3, The authors wrote the MI03920 as MIC9320 incorrectly in many different places.

4, The figure legend in Figure 9C and Figure 8E are the same, but apparently it should be Repo-Gal4 in Figure 9.

Minor points:

1, line 72, the parenthesis is not closed.

2, line 214 and table 1, Rpl11 should be RpL11.

3, line 215, it should be Student’s t-test.

4, line 342, 2b should be 2B.

5, line 1103, p should be italic.
