# Supplementary figures and images for: Drosophila Tet Is Required for Maintaining Glial Homeostasis in Developing and Adult Fly Brains
Source: eNeuro. 2022 Apr 22;9(2):ENEURO.0418-21.2022. doi: 10.1523/ENEURO.0418-21.2022 (PMC9045479; doi:10.1523/ENEURO.0418-21.2022)

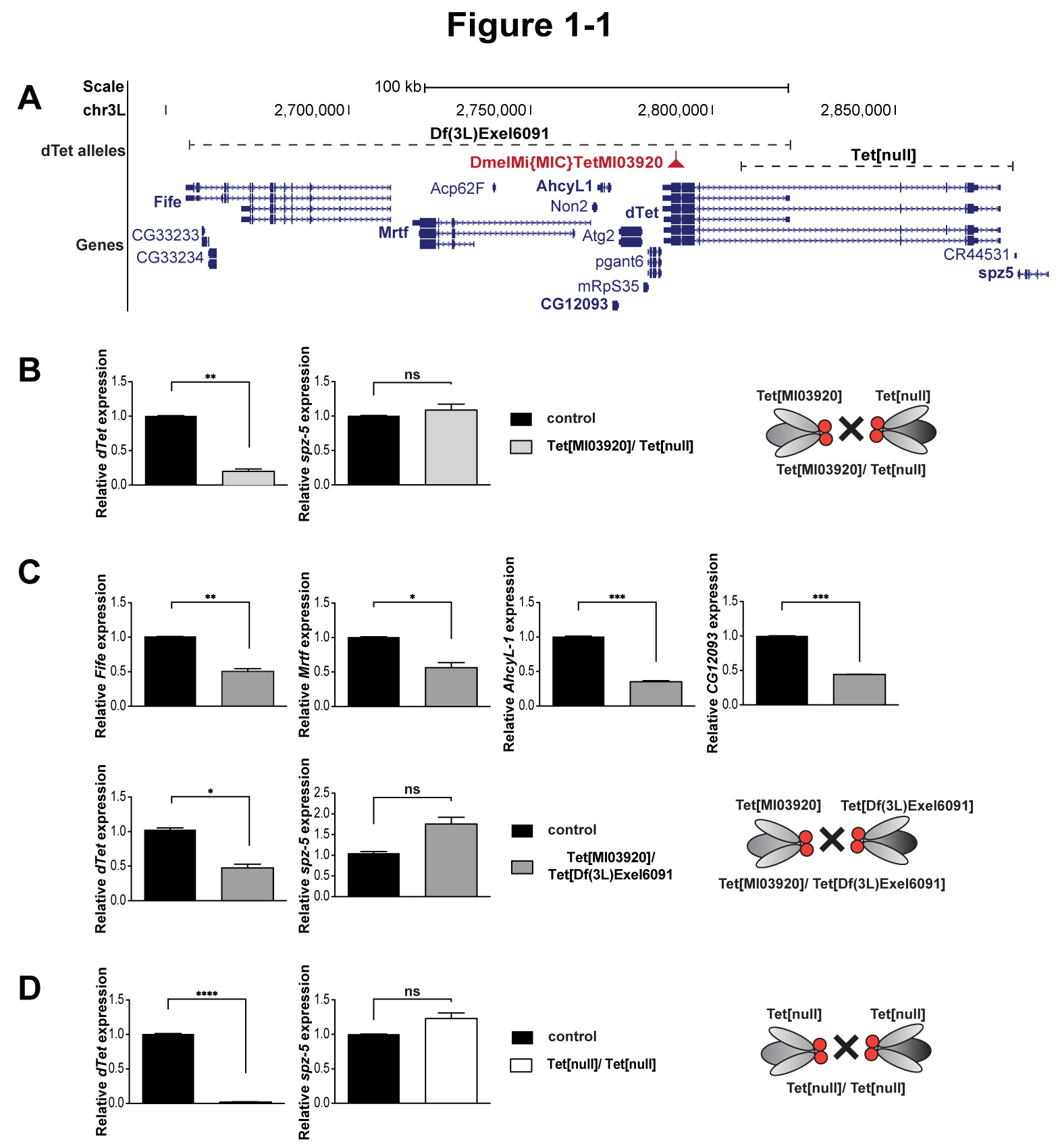

Supplement: Extended Data Figure 1-1 — Changes in expression of dTet and neighboring genes in dTet-deficient flies. A, Gene browser tracks showing the cytogenetic location of Tet[MI03920] insertion, Tet[Df(3L)Excel6091] deficiency, and dTet[null] deletion on Drosophila Chromosome 3 that were used to generate dTet-deficient flies. Note that Tet[Df(3L)Excel6091] deficiency lacks the C-terminal part of dTet gene including the well-conserved catalytical domain as well as several additional genes upstream of dTet including pgant6, CG12093, AhcL1, Mrtf, Fife, etc. Tet[MI03920] fly strain has a MiMIC transposon containing a gene-trap cassette and the yellow+ marker flanked by two inverted bacteriophage ΦC31 integrase attP sites in the last exon of dTet thus interrupting the catalytic domain of all six dTet isoforms. In order to generate dTet-deficient animals, Tet[MI03920] flies were either crossed to Tet[null] deletion (Tet[MI03920]/Tet[null]) or to Tet[Df(3L)Excel6091] deficiency (Tet[MI03920]/Tet[Df(3L)Excel6091]). B, Relative expression of dTet and several adjacent genes including Fife, Mrtf, AhcL1, CG12093, and spz-5 in Tet[MI03920]/Tet[Df(3L)Excel6091] flies. Note that all genes missing in Tet[Df(3L)Excel6091] deficiency show about 50% reduction in expression levels. dTet also showed only 50% reduction in expression level, indicating that Tet[MI03920] insertion allele can produce dTet mRNA. C, Relative expression of dTet and directly upstream located gene spz-5 in Tet[MI03920]/Tet[null] and (D) Tet[null]/Tet[null] flies. Expression level of spz-5 is not affected in Tet[MI03920]/Tet[null] or Tet[null]/Tet[null] flies. Tet[MI03920]/Tet[null] flies showed only reduced levels of dTet mRNA due to the Tet[MI03920] insertion allele, while no dTet mRNA was detected in Tet[null]/Tet[null] flies. Genotypes are as follows: control: wild-type w1118; Tet[MI03920]/Tet[null]; Tet[MI03920]/Tet[Df(3L)Excel6091] and Tet[null]/Tet[null]. Displayed values are mean of two biological replicates (n = 2, 25 larvae per group) eac [file enu-eN-NWR-0418-21-s04.tif]

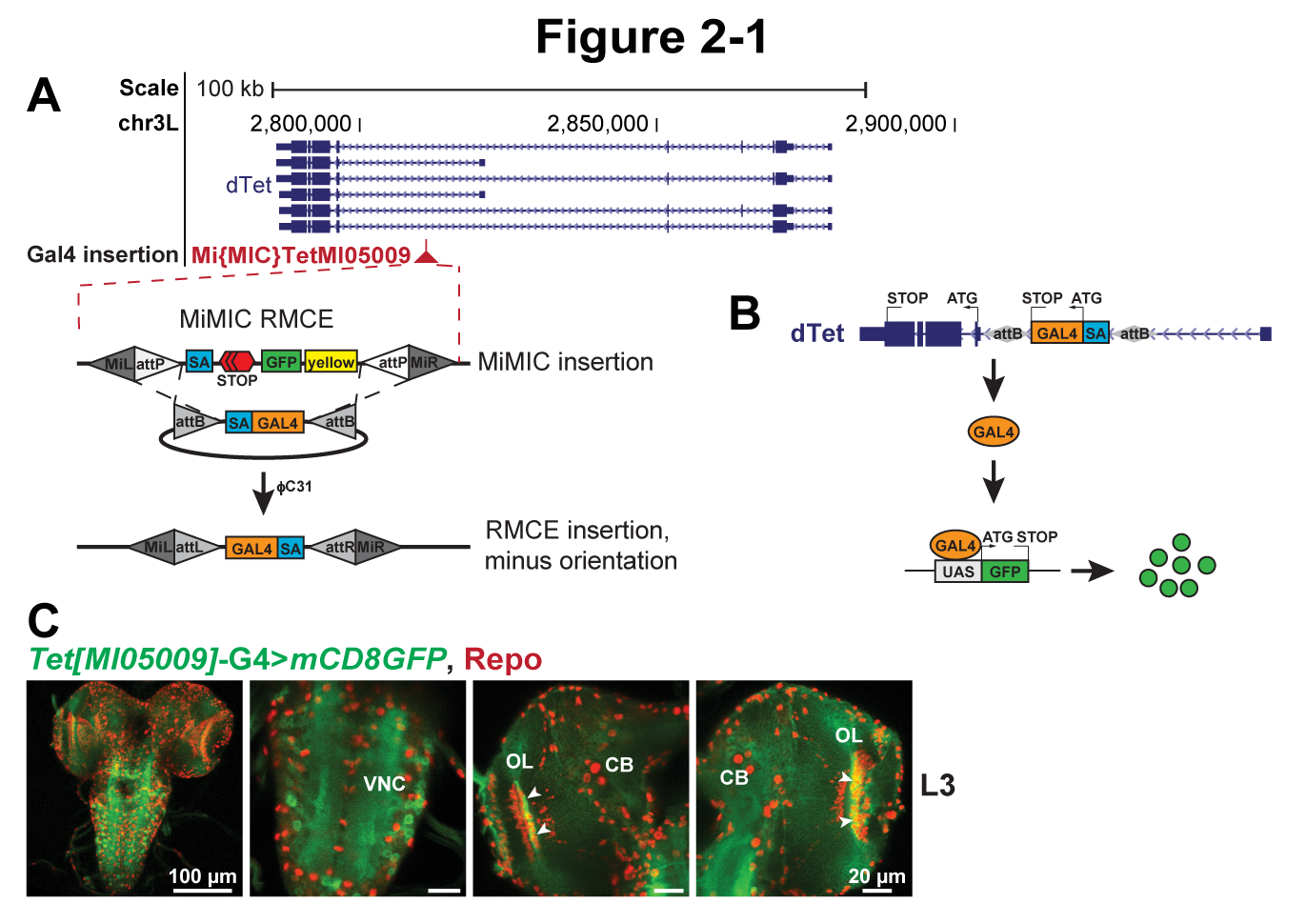

Supplement: Extended Data Figure 2-1 — Generation of Tet[MI05009]-G4 line. A, Genomic location of Gal4 cassette insertion site MI[MIC]TetMI05009 located in the 5′ untranslated region of the short dTet isoforms. MiMIC consists of two Minos inverted repeats (MiL and MiR), two inverted ΦC31 integrase attP sites, a gene-trap cassette consisting of a splice acceptor site (SA) followed by stop codons in all three reading frames and the EGFP coding sequence with a polyadenylation signal (pA), and the yellow+ marker. The sequence between the attP sites was replaced via RMCE with a plasmid containing a Gal4 sequence between two inverted attB sites, resulting in insertion of the Gal4 sequence between attL and attR sites. Recombination can occur in reverse or forward orientation relative to the targeted locus and is shown here in the forward orientation. B, Expression of UAS-GFP using Tet[MI05009]-G4 line results in green fluorescent protein expression in all dTet-expressing cells. C, Immunofluorescence co-staining of whole-mount Tet[MI05009]-G4> UAS-mCD8 GFP larval (L3) brains that express membrane targeted green fluorescent protein through the Tet[MI05009]-G4 driver with anti-Repo (glial cell marker). RMCE, recombinase-mediated cassette exchange; MiMIC, Minos-mediated integration cassette. Download Figure 2-1, TIF file. [file enu-eN-NWR-0418-21-s05.tif]
